# RNA *Cis*-Elements Involved in Animal Virus Stop Codon Readthrough: Stop Codon Context and Downstream RNA Structures

**DOI:** 10.3390/v18080893

**Published:** 2026-08-13

**Authors:** Nobuhiko Kamoshita

**Affiliations:** 1 Department of Biochemistry, Jichi Medical University, 3311-1 Yakushiji, Shimotsuke-shi 329-0498, Tochigi-ken, Japan; nobuhikokamoshita@gmail.com or nobuhiko_kamoshita@urmc.rochester.edu; Tel.: +1-(585)-415-7282; 2 Department of Biochemistry and Biophysics, University of Rochester, Medical Center (URMC), 601 Elmwood Ave., Rochester, NY 14642, USA; 3 Independent Researcher, 1400 Plymouth Ave. S, Rochester, NY 14611, USA

**Keywords:** RNA virus, ribosome, stop codon readthrough, translation termination, translational recoding, *cis*-acting RNA elements, RNA structures, kinetic proofreading

## Abstract

Stop codon readthrough is a noncanonical translation strategy employed by certain RNA viruses, in which a viral termination codon is either decoded by host near-cognate tRNAs or canonically recognized by the class I release factor (RF, eRF1 in eukaryotes). Ribosomal A-site competition between near-cognate tRNAs and eRF can shift decoding toward near-cognate tRNAs, thereby promoting non-canonical decoding events by transiently pausing termination and favoring readthrough. This review focuses on two viral *cis*-elements that modulate readthrough across four viral genera in which this decoding event has been experimentally validated: (i) primary sequences surrounding the stop codon (stop codon context), and (ii) downstream RNA structures. Effects of stop codon context have been observed more broadly in cellular genes, including nonsense suppression in bacteria, with mechanisms including inefficient RF association or tRNA interactions at adjacent sense codons. In eukaryotic systems, interactions with the ribosomal mRNA entry channel have been suggested. Diverse downstream structures, including gammaretroviral pseudoknots and specific structures in alpha- and coltiviruses, further stimulate readthrough in a location- and structure-sensitive manner. This effect has not been consistently observed in chikungunya and triatoviral structures, suggesting a strong dependence on local sequence and structural context. Compared with the larger number of cellular readthrough occurrences that can be detected at low efficiency by ribosome profiling, viral readthrough in mammalian systems is consistently high (>2%). Understanding the interplay between viral RNA elements and host translational machinery, including potential kinetic trapping at the termination codon, provides insights into this unusual elongation mechanism. These findings may have implications for antiviral strategies targeting these RNA elements.

## 1. Introduction

Stop codon readthrough represents an atypical mode of translational elongation. Although a specific termination codon is canonically and efficiently recognized by class I translational release factors (RF1 and RF2 in bacteria and eRF1 in eukaryotes), it can be competitively decoded by near-cognate tRNAs, which typically contain a single codon–anticodon mismatch with the stop codon, resulting in the synthesis of polypeptides of distinct lengths [1,2,3,4,5,6]. The open reading frame preceding the stop codon (ORF1) can either undergo stop codon readthrough to generate a fused ORF1–ORF2 polypeptide or undergo canonical termination to yield the predominant ORF1 product. Viral stop codon readthrough was first described in bacteriophage Qβ [7], Moloney murine leukemia virus (MoMLV) [8], and tobacco mosaic virus (TMV) [9], highlighting its evolutionary conservation across biological kingdoms. Related stop codon readthrough events have subsequently been reported in both prokaryotic and eukaryotic nonviral genes [10,11,12,13,14,15,16,17]. Accumulating evidence from genetic, biochemical, and genome-wide studies indicates that such events may be more widespread and modulated by cellular or environmental stress conditions [18,19,20,21,22,23,24]. Over the past 15 years, various experimental approaches have revealed an increasing number of readthrough events at normal termination codons (NTCs) in higher eukaryotes, including low-frequency events identified through ribosome profiling [25,26,27,28,29,30]. In viruses, nearly twenty eukaryotic virus families isolated from diverse host species use stop codon readthrough to produce primarily coat proteins or replicases [1,3,31,32]. Understanding the molecular events underlying readthrough benefits both basic research and social applications across agriculture, aquaculture, medicine, and zoology. Because viral termination codons can be viewed as positional analogs of premature termination codons (PTCs) in human nonsense mutations—which account for ~11% of hereditary disorders—studying viral readthrough in human cells may provide insights into therapeutic strategies [33,34,35,36,37,38,39].

**Figure 1:**
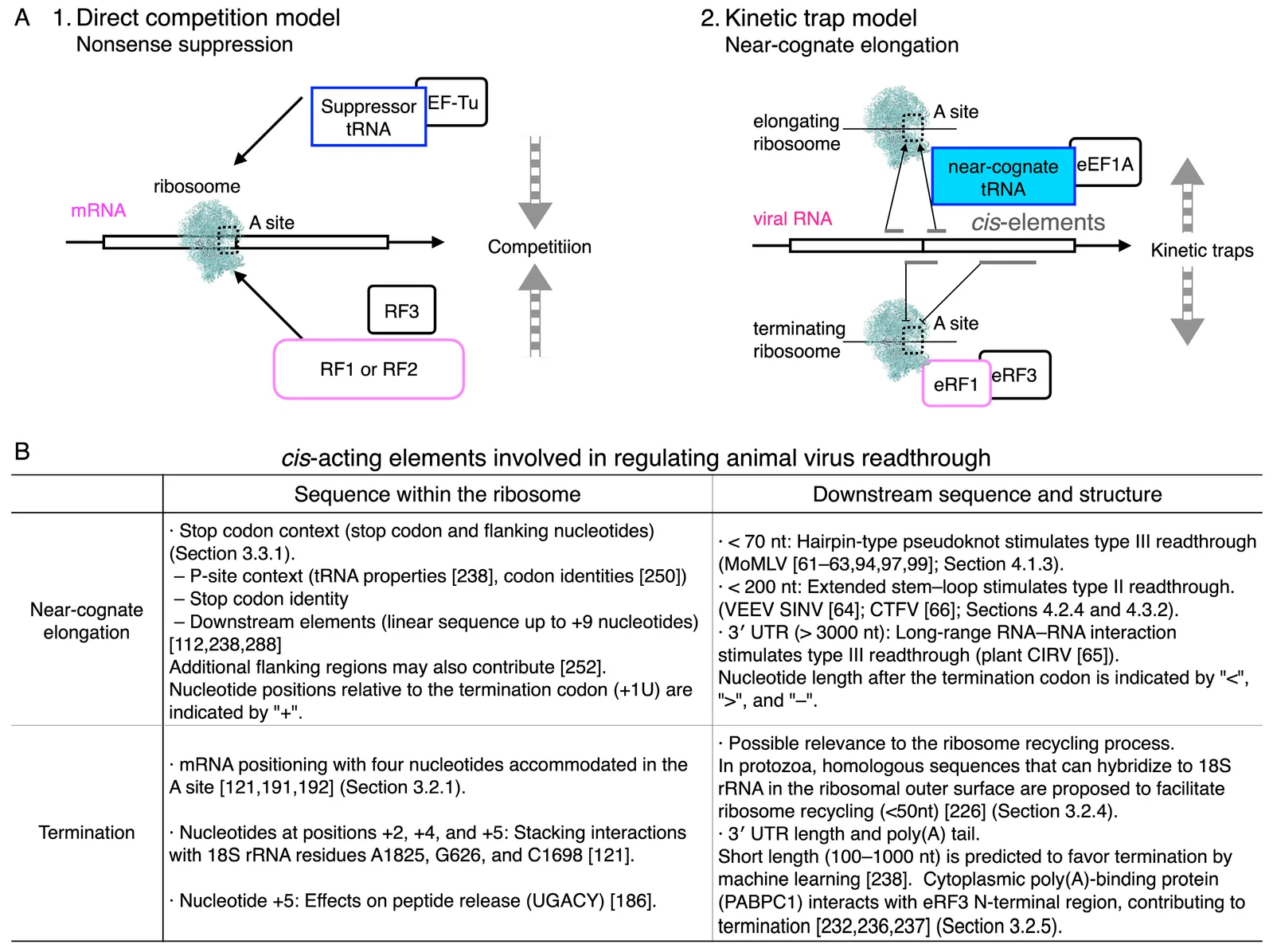
Models and *cis*-acting elements involved in animal virus readthrough. (**A**) Models illustrating the competition between termination and noncanonical elongation at readthrough sites, shown for anticodon-mutant tRNAs (left) and near-cognate tRNAs (right). The direct competition model (left) represents anticodon-dependent nonsense suppression typically observed in bacteria, where nonsense suppressor tRNAs (blue box) carrying mutations in the anticodon allow cognate recognition of the termination codon in the ribosomal A site (dotted rectangle) and directly compete with type I release factors (pink boxes, RF1 and RF2 in bacteria, and eRF1 in eukaryotes). The kinetic trap model (right) represents animal virus readthrough in the absence of mutant tRNAs. In this model, near-cognate tRNAs (shaded blue box), which normally recognize their specific sense codons, decode the stop codon at the readthrough site, supported by *cis*-elements on viral RNAs. Putative kinetic traps on viral RNA *cis*-elements (gray bars) are schematically shown, with arrows indicating the promotion of near-cognate elongation and blunt-ended arrows indicating the inhibition of termination. Given the weak decoding activities of near-cognate tRNAs, multiple traps may form at different sites along the RNA, with some traps partially overlapping, biasing the ribosome toward either continued elongation or termination. Translational GTPases (EF-Tu/eEF1A for elongation and RF3/eRF3 for termination) are represented as black boxes. (**B**) *Cis*-acting elements affecting near-cognate elongation (top) and termination (bottom) are arranged according to their location on mRNA: within ribosomes (left) and in downstream sequences outside ribosomes (right). Reported downstream stimulatory RNA structures and interactions in RNA viruses are listed in the upper right. Related sections are indicated inside parentheses. Virus abbreviations are listed in the section “Representative Viruses and Abbreviations”. Together, these factors create a kinetic trap that modulates the balance between canonical termination and noncanonical elongation at viral readthrough sites.

Readthrough is mediated by elongator tRNAs, which are either mutants—such as nonsense suppressor tRNAs in microorganisms, engineered suppressor tRNAs developed by humans [40,41], or the recently developed anticodon engineered tRNAs (ACE-tRNAs) in mammals [42]—or naturally occurring, including those used in recoding, such as selenocysteinyl, pyrrolysyl, and near-cognate tRNAs [1,11,43,44,45] (Figure 1). In line with the amino acid information incorporated at the readthrough site [46,47], viral readthrough during the normal infectious cycle is considered to use near-cognate tRNAs. In the absence of specific mRNA signals, such as defined nucleotide motifs or RNA structures, elongation at the termination codon by near-cognate tRNAs results in nonsense-to-sense missense errors in eukaryotes. The frequency of such errors is relatively low, on the order of 10^−4^ to 10^−3^ in yeast (*Saccharomyces cerevisiae*) [48], but is generally comparable to or higher than the reported missense error rates at sense codons [48,49,50]. Notably, translational error rates at sense codons are generally lower in eukaryotes than in bacteria, where frequencies of 10^−3^–10^−4^ [51,52] or higher [53,54] have been reported, although substantial codon- and method-dependent variation has also been observed [55,56]. While ribosome profiling has revealed that stop codon readthrough occurs pervasively at NTCs in eukaryotic transcripts, its overall frequency remains low (see the supplementary tables in Ref. [25] and the source data for Figure 6 in Ref. [57]). The efficiency of animal virus readthrough, typically 2–15% under standard mammalian conditions, but higher under certain conditions (see the discussion in Section 2.4), is lower than that reported for nonsense suppression by mutant tRNAs, where efficiencies exceeding 50% have been reported. However, it remains markedly higher than translational errors or low-frequency readthrough events at NTCs. This review focuses on viral RNA *cis*-elements involved in readthrough, providing insights into how viral strategies exploit the host translational apparatus while distinguishing this recoding from ordinary translational errors.

As one type of recoding [58,59,60], stop codon readthrough is associated with two classes of RNA elements: (i) local nucleotide sequences flanking the termination codon, now frequently called the ‘stop codon context’, which has recently been intensively studied in mammalian cellular genes, and (ii) downstream stimulatory RNA structures. For the latter, a gammaretroviral hairpin-type (H-type) pseudoknot structure [61,62,63] and other forms such as extended stem–loops [64], long-distance RNA–RNA interactions [65], and a forked structure [66] have been identified. Meanwhile, additional downstream extended stem–loops have been reported in chikungunya virus (CHIKV) [67,68] and Plautia stali intestine virus (PSIV) [69]. While the stimulatory effects are absent in PSIV and remain unconfirmed or untested in CHIKV, their presence highlights the need for a comprehensive evaluation of downstream elements in viral readthrough.

**Figure 2:**
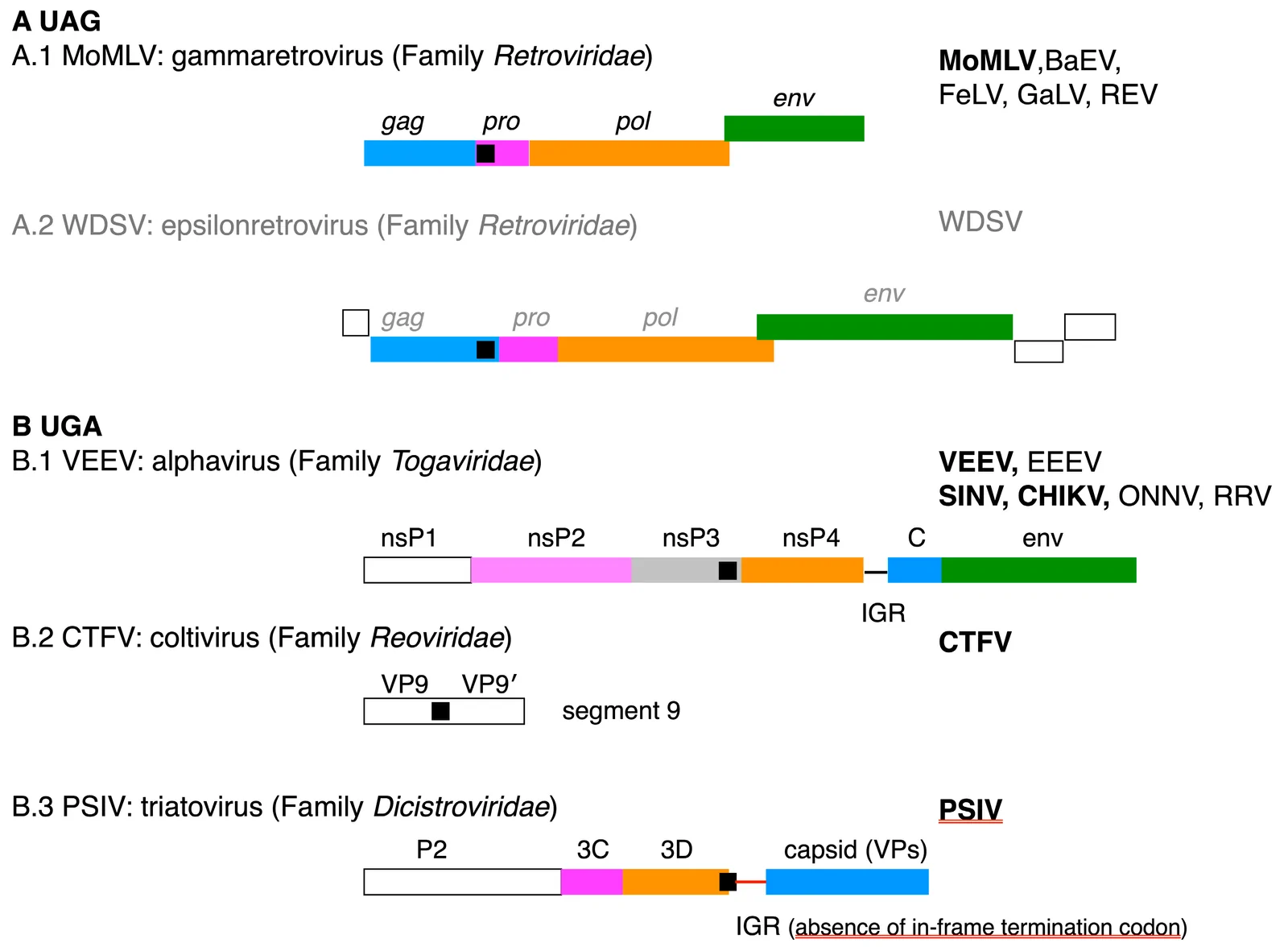
Gene structures and stop codon readthrough sites in animal viruses. (**A**) UAG and (**B**) UGA readthrough sites are shown as black squares. Representative strains are shown on the right (see Representative Viruses and Abbreviations); experimentally investigated species are shown in bold. Blue, nucleocapsid; magenta, protease; orange, polymerase; green, envelope protein; white and gray, others. Intergenic regions (IGRs) are shown as lines; the PSIV readthrough-associated IGR is shown in red. In this figure, PSIV P2 includes proteins corresponding to 2A–2C, 3A, and 3B (VPg).

**Figure 3:**
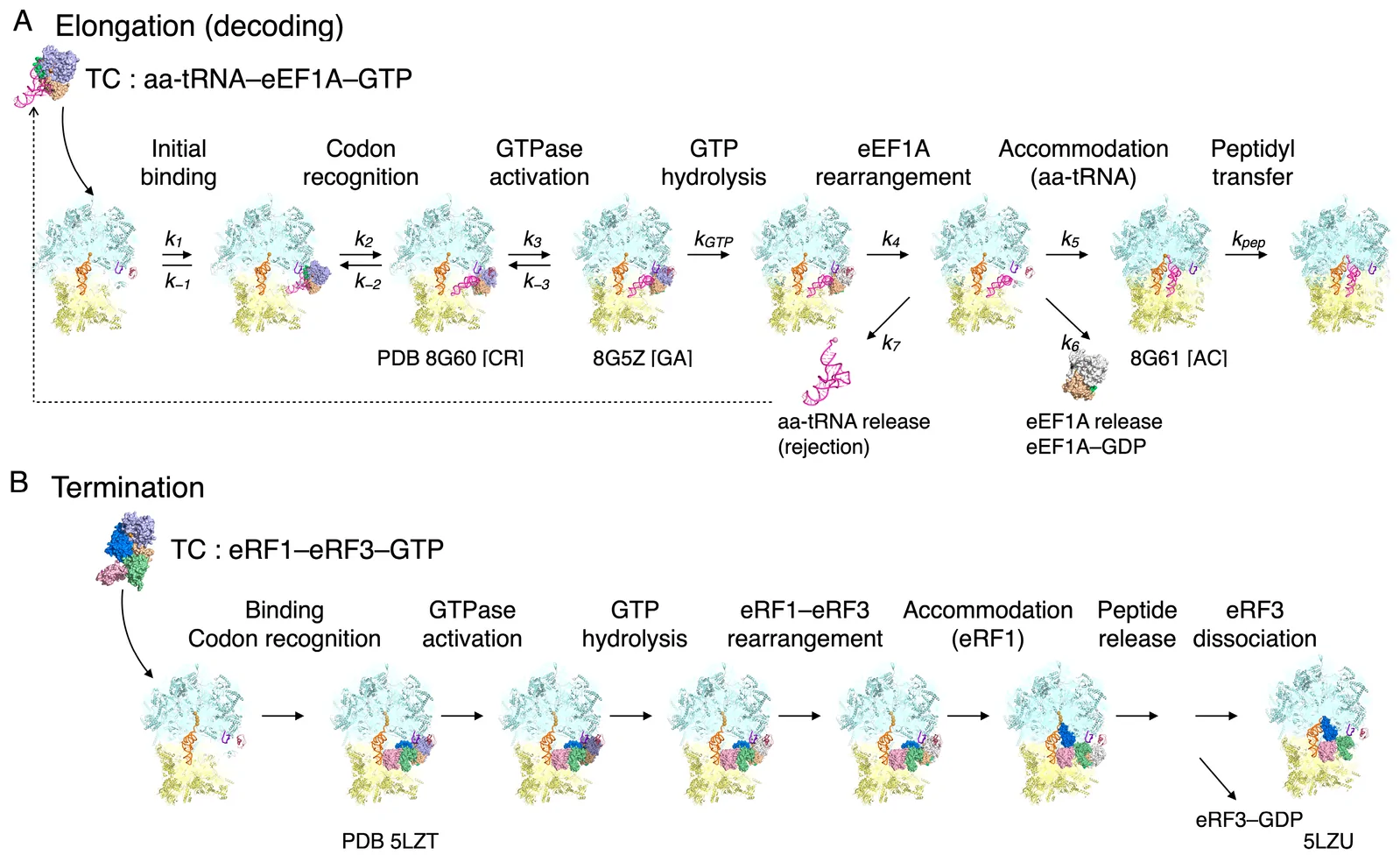

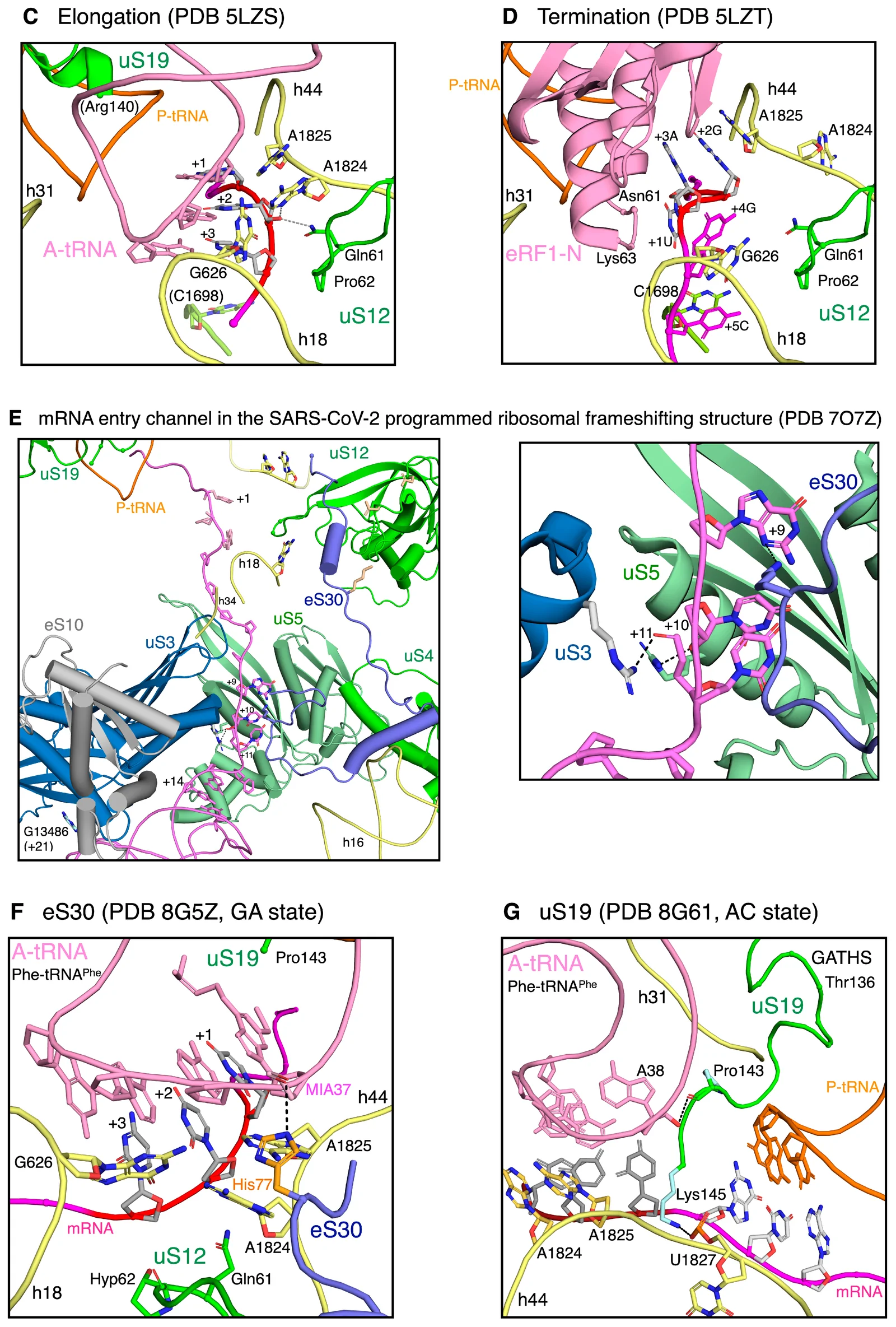
Structural and mechanistic overview of eukaryotic translation. (**A**,**B**) Schematic representation of elongation (decoding) and termination pathways. (**A**) Expected mechanisms for selecting cognate and rejecting near-cognate tRNAs during decoding are shown schematically, with associated rate constants adopted from bacterial kinetic and structural studies [51,114,118] and corresponding mammalian cryo-EM structures [119]. (**B**) Mechanisms underlying stop codon recognition and peptide release during termination are summarized from eukaryotic studies [120], with the corresponding cryo-EM structures [121]. Each panel illustrates representative molecular events and factors involved in decoding and termination. Solid arrows indicate transitions within the canonical decoding pathway, whereas dotted arrows in panel A indicate re-formation of the ternary complex (TC) after rejection. In both panels, the GTPase domains 1–3 in the TC (eEF1A in (A); eRF3 in (B)) are colored light blue, lime green, and wheat (light yellow–orange), respectively, with the catalytic histidine in domain 1 highlighted in bright orange; domain 1 is shown in white in the GDP-bound form. eRF1 domains are colored pink (N), marine blue (M), and pale green (C). Amino acids in the nascent chain (the single fMet residue in A and four arbitrarily selected residues in (B)) are depicted as orange spheres, indicating the location of P-site tRNA during peptidyl transfer and peptide release. The sarcin–ricin loop (SRL) and a portion of P0 are depicted as a purple ribbon and a raspberry cartoon, respectively. Ribosome, factor, and tRNA structures are derived from structural studies [119,121,122,123,124], and PDB accession numbers for ribosomal studies are indicated. TC, ternary complex; CR, codon recognition state; GA, GTPase activation state; AC, accommodated state. (**C**,**D**) Decoding center interactions viewed from the small subunit body side (uS12 indicated) during canonical codon recognition. The canonical recognition of mRNA codon (backbone in red, base–sugar in gray) by the decoding factor in the A site (pink: (C) cognate tRNA and (D) eRF1 N domain) and by selected 18S rRNA nucleotides (pale yellow). The relevant amino acids on uS12 and eRF1 are shown in stick and sphere representation. Structural data are from PDB entries (C) 5LZS and (D) 5LZT [121] employing ribosomes from rabbit reticulocyte lysate (RRL). Both views are shown in a similar orientation and scale using PyMOL (version 3.1.4.1; Schrödinger, LLC, New York, NY, USA). Portions of molecules, including eS30, A-site tRNA stem–loops not involved in codon pairing, and the eRF1 M and C domains, were hidden for clarity. The RNA backbones, including mRNA (magenta), A-site tRNA (pink), and portions of 18S rRNA (pale yellow), are shown as cartoons. Note that in ribosomes terminating at the UGAGC sequence, the positioning and conformation of the mRNA differ from those in elongation (C), and the +5U nucleotide is recognized by 18S rRNA C1698 (light green) through a stacking interaction (D). The C-terminal residues of uS19 (up to Lys145) are not visible; the most C-terminal residue shown is Arg140 in C (sphere), whereas the density in D is resolved only to Pro131. In panel C, the hydrogen-bonding network surrounding the +2 nucleotide is indicated by gray dashes. Water-mediated interactions are not shown. A1824 N3 is located 2.9 Å from the 2′-hydroxyl oxygen (O2′) atom of mRNA +2 (short gray dash), consistent with an A–minor interaction. Gln61 NE2 is 3.2 Å from the O2′ of mRNA +2 (long gray dash), forming part of a hydrogen-bonding network involving uS12. (**E**) Viral mRNA–ribosome interactions around the mRNA entry channel. 18S rRNA helices h18 (G625–A629) and h34 (C1501–U1503), which together form the mRNA entry channel latch (constriction), are shown along with ribosomal proteins, uS3 (deep blue), uS5 (pale green), eS10 (gray), and eS30 (slate), which surround the downstream region of the SARS-CoV-2 frameshifting mRNA (pink). RNAs and proteins are shown as cartoons. The arginine and lysine residues of uS3, uS5, and eS30, near the mRNA entry pore and potentially interacting with viral RNAs, are shown as sticks. uS3 Arg117 NH1 is positioned 2.8 Å from the phosphate oxygen (OP1) atom of mRNA +11, consistent with a close contact, whereas uS5 Arg152 NH1 is 3.4 Å from O2′ of mRNA +10 and eS30 Lys125 NZ is 3.7 Å from N3 of mRNA G+9, suggesting weaker interactions [125]. On the ribosomal outer surface, Loop 1 of the viral RNA, including nucleotide G13486 (+21 from the A-site codon, side chain shown as sticks in pale cyan), makes close contacts with uS3 and lies adjacent to eS10. Ribosomal helix h16 (pale yellow) approaches the pseudoknot S1 region, but interactions between h16 and S1 are not shown; only their relative positioning is indicated. Ribosomal nucleotides and protein residues surrounding the decoding center are shown in (C,D). Representative residues in proximity to the DHX29 double-stranded RNA-binding domain (dsRBD) [126] are highlighted in wheat: uS12 (Asn92 and Glu95) and eS30 (Lys92). The ribosome context is highlighted by uS12 and uS19 (green), with uS4 included as an additional reference; its role in mRNA entry in eukaryotes remains unclear. In this structure (PDB 7o7z), no molecule occupies the A site. The mRNA nucleotide positions are numbered relative to the A-site codon. The pseudoknot starts at position +14 in this structure. Several ribosomal proteins are omitted for clarity. (**F**,**G**) Stabilization of the canonical decoding geometry by eS30 and uS19 C-terminal tail. The canonical decoding of a sense codon by cognate *E. coli* Phe-tRNA^Phe^ at the A site of the human ribosome is shown in the GTPase activation ((F), GA) and accommodated ((G), AC) states [119]. These canonical geometries are stabilized by eukaryote-specific components, including eS30 (F) and the C-terminal extension of uS19 (G), with all colors and residue representations following (C). (**F**) The N-terminal His77 residue of eS30 (orange, the third residue from the N-terminus) participates in a hydrogen-bond network in the A–minor groove that stabilizes the canonical decoding geometry. NE2 of His77 is 3.0 Å from OP1 of A-tRNA residue MIA37 (MIA, also known as ms^2^i^6^A, 2-methylthio-*N^6^*-isopentenyladenosine). uS12 residue 62 (Hyp62) is also shown. (**G**) The C-terminal Pro143 and Lys145 residues of uS19 (pale cyan) interact with the backbones of the A-site tRNA anticodon loop and 18S rRNA h44, respectively. The carbonyl oxygen of Pro143 lies 3.2 Å from O2′ of A-site tRNA A38, while the NZ of Lys145 is positioned 3.0 Å from OP1 of U1827 in 18S rRNA. These interactions stabilize the canonical decoding geometry by reinforcing both A–minor interactions and the positioning of the A-site tRNA. Structures are from PDB 8G5Z ((F), GA state) and 8G61 ((G), AC state). Hyp62, hydroxylated Pro62 of uS12; MIA, ms^2^i^6^A.

This review focuses on stop codon readthrough in animal viruses, which has been primarily studied in mammals. Given that only six viral species have been intensively investigated (Figure 2), the distribution of homologous sequences is presented first, followed by an overview of readthrough events. I next outline potential substages (Figure 3A,B) where kinetic traps (a concept proposed to characterize recoding [70]; see Figure 1A) may operate alongside normal translational elongation and termination. Drawing on recent insights from cellular gene assays and ribosome structural studies, I highlight how viral RNA features—stop codon context and downstream structures—can contribute to these traps (Figure 1B). The role of readthrough in each viral genus is addressed, with emphasis on the features of genus-specific downstream structures. Differences in their effects and potential explanations are discussed, followed by directions for future investigation. For broader aspects of viral recoding and general principles of stop codon readthrough, readers are referred to relevant reviews [1,3,31,32,39,71].

**Table 1:** Viruses and RNA elements relevant to animal virus stop codon readthrough.

Investigated Virus		Stop-Codon Flanking Sequence			Downstream Structure			Reference	NCBI RefSeq	
Genus	Virus	Beier–Grimm Type	Nucleotide [−12 to +12] ^a,b^ Amino Acid [−4 to +4] ^b^	Stop Codon Position ^b^	Fold Type	Start (nt) ^c^	Size (nt)		Accession	Length (nt)
*Gamma* *retrovirus*	**MoMLV**	III	ACC CUA GAU GAC UAG GGA GGU CAG GGU Thr Leu Asp Asp Xaa Gly Gly Gln Gly	1971	H-type pseudoknot	+12	50	[61,62,63]	NC_001501	8332
*Epsilon* *retrovirus*	**WDSV**	III	ACA UAU CCU GCA UAG GAU CCA AUU GAC Thr Tyr Pro Ala Xaa Asp Pro Ile Asp	2546	ND	–	–	–	NC_001867	12,708
*Alphavirus*	**VEEV** subtype ID	II	GCA CAA CAA CAA UGA CGG UUU GAC GCG Ala Gln Gln Gln Xaa Arg Phe Asp Ala	5682	Extended stem–loop	+12	126	[64]	NC_001449	11,444
	**SINV**	II	AGG ACU GAA UAC **UGA C**UA ACC GGG GUA Arg Thr Glu Tyr Xaa Leu Thr Gly Val	5748	Extended stem–loop	+16	177	[64,72]	NC_001547	11,703
	**ONNV**	II	GAU GAA GAG UUA UGA CUA GAC AGA GCA Asp Glu Glu Leu Xaa Leu Asp Arg Ala	5765	ND	–	–	[73]	NC_075006	11,822
	**CHIKV**	II	GAC GAC GAG UUA CGA* CUA GAC AGG GCA	5645 *	Extended forked stem–loop	+9	133	[67,68]	NC_004162	11,826
			Asp Asp Glu Leu Arg* Leu Asp Arg Ala			+13	127	[68]		
*Coltivirus*	**CTFV**	II	AAG GGC UGC UGU UGA CGG UGU UGG CCU Lys Gly Cys Cys Xaa Arg Cys Trp Pro	1052	Extended forked stem–loop	+12	78	[66]	NC_004180	1884
*Triatovirus*	**PSIV**	II	GAA GAA GAA AGC **UGA CUA** UGU GAU CUU Glu Glu Glu Ser Xaa Leu Cys Asp Leu	6004	Extended stem–loop	+2	68	[69]	NC_003779	8797
*Tobamovirus*	**TMV**	I	GCA GGA ACA CAA **UAG CAA UUA** CAG AUU Ala Gly Thr Gln Xaa Gln Leu Gln Ile	3417	NA	–	–	[9,74,75]	NC_001367	6395
*Qubevirus*	**Qβ**	n.c.	AAU CCG GCG UAC UGA ACU GCA CUU CUU Asp Pro Ala Tyr Xaa Thr Ala Leu Leu	1742	ND	–	–	[7]	NC_001890	4215

^a^ Nucleotides reported in at least one study to contribute to readthrough efficiency are bolded, whereas those involved in the formation of the downstream structure are underlined. ^b^ The asterisk for CHIKV indicates the CGA (Arg) codon in the NCBI reference sequence, which is replaced with a UGA stop codon in most strains. ^c^ +1 = first nucleotide of the readthrough stop codon. Two nucleotide ranges for alternative CHIKV conformations (Section 4.2.4) are indicated in the upper and lower lines. Abbreviations: H-type pseudoknot, hairpin-type pseudoknot; NA, not applicable (no downstream stimulatory structure required); n.c., not classified (Beier–Grimm type not applicable to non-eukaryotic viruses); ND, not determined; RefSeq, reference sequences (NCBI database); –, not available.

### Representative Viruses and Abbreviations

BaEV, baboon endogenous virus; CHIKV, chikungunya virus; CIRV, carnation Italian ringspot virus; CTFV, Colorado tick fever virus; EEEV, eastern equine encephalitis virus; FeLV, feline leukemia virus; FI 4184 b, bacteriophage strain FI 4184 b; GaLV, gibbon ape leukemia virus; MoMLV, Moloney murine leukemia virus; ONNV, o’nyong-nyong virus; PSIV, Plautia stali intestine virus; Qβ, bacteriophage Qβ; REV, reticuloendotheliosis virus; RRV, Ross River virus; SFV, Semliki Forest virus; SINV, Sindbis virus; TMV, tobacco mosaic virus; VEEV, Venezuelan equine encephalitis virus; WDSV, walleye dermal sarcoma virus; WEEV, western equine encephalitis virus.

## 2. Overview and Distribution of Animal Virus Stop Codon Readthrough

### 2.1. Classification

Viral stop codon readthrough has been classified into three types (Beier and Grimm classification) based on the conserved nucleotide sequence beginning from the readthrough termination codon [1]. Type I is characterized by a nonanucleotide sequence UAGCAAUUA (with the stop codon underlined), type II is characterized by hexanucleotide sequences UGACGG or UGACUA, and type III is characterized by sequences UAGGGV (V=A, C, G), UAGGUA, or UAGGAU. All three types are present in plant viruses, while only types II and III are present in animal viruses.

Experimental evidence of animal virus readthrough has been obtained from the following four genera (Table 1): *Gammaretrovirus* (family *Retroviridae* with hexanucleotide UAGGGA [61,63,76]), *Alphavirus* (family *Togaviridae* with UGACGG in Venezuelan equine encephalitis virus (VEEV) [64] and with UGACUA in other species [64,67,68,72,73]), *Coltivirus* (family *Reoviridae* with UGACGG [66]), and *Triatovirus* (family *Dicistroviridae* with UGACUA [69]) (Table 1 and Figure 2). Not all viral readthrough sequences have been classified according to the Beier and Grimm classification; for instance, the hexanucleotides in phage Qβ or FI 4184 b in the family *Fiersviridae* in bacteria are UGAACU or UGAGCR [32,77], respectively. Viral readthrough can be stringently classified into seven or more groups based on the similarities of downstream nonanucleotide sequences [32]. A key feature of Beier and Grimm classification is that it reflects the underlying mechanism (Section 2.5).

### 2.2. Distribution

Viral replication strategies are expected to be conserved within the same genus. Therefore, more than 50 animal viral species are anticipated to incorporate stop codon readthrough (Table 2). Most importantly, stop codon readthrough is a criterion for classifying the post-transcriptional expression of the *pol* gene in gamma- and epsilon-retroviruses. Representative gammaretroviruses include oncogenic murine leukemia viruses, feline leukemia virus (FeLV), gibbon ape leukemia virus (GaLV), Koala retrovirus (KoRV), and avian reticuloendotheliosis virus (REV) (Table 2). Exogenous retroviruses can both become endogenized in their host genomes and pose a risk to other species through cross-species transmission, as suggested by observations in KoRV [78] and comparative viral genomic analyses of REV [79]. Full-length gammaretroviral endogenous retroviruses with stop codon readthrough sites have been identified in multiple mammalian lineages, including bats, deer, felines, koalas, primates, rodents, swine, and killer whales (Table 2).

Viruses from all three other genera replicate in arthropods. Two of them, *Alphavirus* and *Coltivirus*, are arboviruses that are transmitted by arthropods and cause disease in humans and other mammalian hosts. Alphaviral strains are further classified into seven or more antigenic complexes based on serological relationships (Table 2). The VEE, Semliki Forest (SF), and western equine encephalitis (WEE) complexes each include multiple species or their subtypes that are important pathogens in human or veterinary medicine. Among them, reflecting several recent outbreaks of CHIKV, more than 5000 full-length CHIKV isolates have been registered in the NCBI GenBank, highlighting the medical threat posed by this virus. More than 100 isolates have also been sequenced and reported in VEEV, eastern equine encephalitis virus (EEEV), SINV, and Ross River virus (RRV). The remaining genus, *Triatovirus*, belongs to the family *Dicistroviridae*, a group of viruses previously called insect picorna-like viruses that cause acute lethal infection in their natural invertebrate hosts. Two species of this genus, PSIV and Triatoma virus (TrV), have downstream in-frame ORFs longer than 180 nucleotides (nt) extending beyond the ORF1 termination codon and spanning the intergenic region (IGR) without in-frame termination codons. Thus, the range of hosts and spectrum of pathogenesis of viruses that incorporate stop codon readthrough are broad.

### 2.3. Significance (Nonsense-to-Sense Codon Mutant Viruses)

Early reverse-genetic studies targeted stop codon readthrough sites in MoMLV and SINV. The consequences of nonsense-to-sense codon substitutions differ among viruses. MoMLV CAG mutants cannot replicate because of defects in Pr200*^gag–pol^* (ORF1–ORF2 fusion product) processing and virion assembly [81,82], and replication defects for AAG MoMLV were independently confirmed [83]. SINV sense-codon substitution mutants (UCC, UGG, and CGA) can replicate, albeit with delayed RNA synthesis, which can be largely compensated for by increasing the multiplicity of infection (MOI) [84]. Comparable delays in RNA synthesis are observed for an independent set of sense-codon substitutions (GCA, UGU, and CGC) at lower MOIs, which are compensated for by cultivation at 28 °C to restore balanced polyprotein processing [85]. These findings indicate that the advantage of the SINV opal termination codon (UGA) lies in maintaining balanced polyprotein processing.

Thus, the viral strategy of using a stop codon instead of sense codons reflects replication demands: (i) enabling proper polyprotein processing and (ii) producing an optimal amount of polymerase. Normal termination at the readthrough site results in partial processing of viral polyproteins, whereas near-cognate elongation enables the production of the polymerase required in limited amounts. These dual mechanisms can provide advantages over simple sense-codon elongation. In alphaviruses that alternately replicate in vertebrate and arthropod hosts, UGA readthrough is advantageous in vertebrate hosts but not absolutely required [85]. In some alphavirus–arthropod combinations, readthrough-mediated polymerase may not be sufficient for optimal replication. In such cases, mutation to a sense codon can represent a viral strategy to enhance replication. Sense-codon isolates have been identified in members of the SF complex and in SINV, as well as in two other alphavirus species (Section 4.2.3 and Table 2).

For certain viruses, such as CFTV and PSIV, the biological significance of stop codon readthrough remains unclear.

### 2.4. Experimental Evidence of Animal Virus Readthrough

Before the advent of reverse genetics, readthrough was observed on sodium dodecyl sulfate–polyacrylamide gel electrophoresis (SDS–PAGE) as C-terminal extensions of viral translation products [7,8,9,86,87]. The absence of alternative mechanisms such as frameshifting, which can now be easily ruled out by mRNA sequence analysis, was simultaneously confirmed by the use of stop-codon-specific nonsense suppressor tRNAs [7,8,9,11]. Experimental detection of extended fusion polypeptides remains important, particularly because related mechanisms, such as translational reinitiation, are naturally coupled to termination and can enable the translation of downstream ORFs in eukaryotes under certain conditions [88,89].

Publications reporting animal virus readthrough are listed in Table 3. While three of the four viral families employ insects as hosts, all four have been mainly investigated in mammalian translation systems. Commercially available rabbit reticulocyte lysate (RRL) and the human embryonic kidney 293 (HEK293) cell line, or its derivatives, are used in most publications. The availability of defined SP6- or T7-derived transcripts facilitated the identification of *cis*-acting elements [61,62,63,72] and systematic evaluation of exogenous factors such as tRNAs involved in stop codon readthrough [75,90].

**Table 3:** Experimental data on stop codon readthrough in animal viruses.

Virus	Assay Construct ^a^	Study Region ^b^ (Core Region)	Cultured Cells ^c^	Cell-Free Systems	Efficiency or Equivalent ^d^	Notes	Reference
MoMLV +MoMSV	Full-length constructs	35S RNA	–	L, HeLa RRL	Product ratio 1:50 ~2% (RRL) ^d^		[86] [8]
Rauscher MLV	Full-length constructs	35S RNA virion proteins	JLS-V16 ^e^	JLS-V16 ^e^ extracts	3–5% (estimated in Discussion)		[87]
MoMLV FeLV	Pr65*^gag^* cleavage	PR amino acids	JLS-V16 ^e^	HTG-2 ^e^ extracts	4–10% (cited in Introduction)	Proposed tRNA^Gln^ misreading	[46] [47]
MoMLV	Viral proteins SP6 transcripts	*gag–pol* to SalI/HindIII	– CHO	RRL (Promega)	~5% (estimated)		[90] [91]
MoMLV	Leader– protein A	−6 UAG +60	–	RRL (Promega)	~5% (cited in Introduction)		[92]
MoMLV	Viral proteins SP6 transcripts	*gag–pol* to BamHI	–	RRL	– ^d^	Basal S1 not restored	[76]
MoMLV	CAT–lacZ T7 transcripts	−27 UAG +60 (−15 UAG +60)	–	RRL WGE (Promega)	2.5% RRL [62]		[62,63]
MoMLV	CATΔ–lacZ	−6 UAG +60	–	RRL (Promega)	Product ratio 1:70 ~1.4% (RRL) ^d^		[61]
MoMLV	rLuc–fLuc [93]	−13 UAG +63	HEK293	RRL (Promega)	3.7% HEK293 8.8% RRL		[93]
MoMLV	CAT–lacZ [62]	−15 UAG +71	–	RRL	Readthrough (%) ^d^ 68 (100%); 57 (25%)		[94]
MoMLV	rLuc–fLuc [93]	−13 UAG +63	HEK293T	–	4.2% HEK293T	Increases to up to 9% by RT MoMLV	[95,96]
MoMLV	rLuc–fLuc	−13 UAG +69	HEK293A	RRL (Ambion)	~7.5%	pH 7.1	[97]
MoMLV	rLuc–fLuc [93]	−13 UAG +63	11 cell lines ^f^	–	3 to ~12%	Increases to ~9% by uL4 (2.3-fold) ^f^	[98]
MoMLV	rLuc–fLuc	100 bp fragments	HEK293T Rat2	–	7% HEK293T 9% Rat2		[99]
MoMLV	Infected-cell transcripts	Entire viral transcriptome	Rat2	–	6–8%	Values from ribosome profiling	[100]
MoMLV	rLuc–2A–2A–fLuc [101]	−13 UAG +96	HEK293T U-87 MG	–	Very similar to CHIKV (below)		[67]
VEEV	rLuc–fLuc	−15 UGA +159	HEK293	TNT RRL (Promega)	7.6% HEK293 2.9% (RRL) ^d^	No stem–loop: 0.8% No stem–loop: 0.2%	[64]
SINV	Full-length virus	nsP3 and nsP4	CEF C7-10	–	Reduced in C + 4U mutant (NQ) ^d^	Host-dependent effects	[102]
SINV	CATΔ–4D4 epitope	0 UGA +9 (0 UGA +4)	–	RRL (Promega)	~10 ± 1% RRL ^g^ (Fig. 2 ^h^)	Downstream structure not included	[72]
SINV	rLuc–Luc	−15 UGA +207	HEK293	TNT RRL (Promega)	6.4% HEK293 1.6% (RRL) ^d^	No stem–loop: 2.0% No stem–loop: 0.4%	[64]
SINV	rLuc–fLuc [93]	Downstream structure (−) ^b^	HEK293A	–	3% HEK293A	Increases to ~8% by uL4	[98]
SINV	nsP3/nLuc–nsP4/fLuc	With RTE	Vero C6/36	–	15% Vero 24% C6/36 (28 °C)	No RTE: 10% No RTE: 10%	[85]
CHIKV	rLuc–2A–2A–fLuc [101]	−10 UGA +141	HEK293T U-87 MG	–	7.0–7.1% HEK293T 13.4–13.7% U-87 MG	Elongation efficiency at sense codon ~90% ^i^	[67]
CHIKV	Sense-to-nonsense mutant	replicon RNA (EU224268)	BHK-21 C6/36 ^j^	–	Replication reduced in UAG, markedly reduced in UAA (Fig. 3 ^h^)	3x stem-disrupting (+15, +18, +21): minimal effect on replication (Fig. 5 ^h^)	[68]
CTFV	rLuc–fLuc	−16 UGA +291 (−16 UGA +89)	HEK293T	TNT RRL ICE (Promega)	2.0% 293T 3.9% RRL 3.1% ICE		[66]
PSIV	rLuc (humanized)–fLuc	−150 UGA +189 (0 UGA +6)	COS-1 HEK293	RRL (Promega) Sf21 extract ^k^	7.9% COS-1 8.6% HEK293 5.0% (RRL) ^d^	Structure not required	[69]

Listed, in the order of reporting, are the investigated virus, assay construct, reported range (regions employed and core elements required for readthrough efficiency), experimental system (cultured cells and/or cell-free systems), and readthrough efficiency. Abbreviations: ICE, insect cell extract (Sf21); PR, MoMLV protease; RRL, rabbit reticulocyte lysate; RT, MoMLV reverse transcriptase (a downstream product of readthrough); RTE, readthrough element; TNT, Promega transcription/translation–coupled in vitro protein expression system; WGE, wheat germ extract. For virus abbreviations, see the “Representative Viruses and Abbreviations” section. ^a^ Reporters (alphabetical order): CAT (chloramphenicol acetyl transferase); fLuc (firefly luciferase); lacZ (β-galactosidase); nLuc (NanoLuc); rLuc (*Renilla* luciferase). 2A, self-cleaving peptide for polycistronic expression. Reporter constructs are generally designated by the genes or reporters immediately flanking the test sequence (e.g., CAT–lacZ). For CAT-based assays, an N-terminal fusion to protein A was used in Refs. [62,63,94], whereas a C-terminally truncated form (CATΔ) was employed in Refs. [61,72]. The pSGD system, a 2A peptide-based expression vector [101], has been applied in several studies, including CHIKV [67]. ^b^ Nucleotide positions are indicated relative to the stop codon, with the first nucleotide (U) designated as +1 and the adjacent upstream nucleotide as −1. Parentheses indicate experimentally defined core *cis*-elements within the analyzed region. (−) indicates that the downstream structure was excluded from the analyzed construct. ^c^ Cell lines: 293, 293A, 293T, HEK293 (human embryonic kidney); BHK–21 (baby hamster kidney fibroblasts); C6/36 (*Aedes albopictus* mosquito embryos/larvae); C7-10 (*Aedes albopictus larvae*); CEF (chicken embryonic fibroblasts); CHO (Chinese hamster ovary); COS-1 (African green monkey kidney); HeLa (human cervical carcinoma); L (mouse fibroblasts); Rat2 (rat fibroblasts); U-87 MG (human malignant glioblastoma); Vero (African green monkey kidney). ^d^ Unless otherwise indicated, values were measured for wild-type sequences. Readthrough (%) represents the fraction of readthrough products relative to total (readthrough + terminated), without normalization to a sense control. Values estimated from reported product ratios are indicated as calculated readthrough percentages. Assay systems are indicated in parentheses when specified. NQ indicates that a qualitative effect was reported but not quantified. “—” indicates that no readthrough data were reported. ^e^ JLS-V16, Swiss mouse embryo fibroblasts chronically infected with Rauscher murine leukemia virus; HTG-2, hamster cells transformed with Gazdar murine sarcoma virus; FeLV was prepared from feline lymphoblasts as described in Ref. [47]. ^f^ Cell lines used: 293A, 293T, BHK-21, HeLa, and Rat2 cells, as well as A549 (human lung carcinoma), BALB/c-derived fibroblasts, La-T (designation as in the original report), NIH3T3 (mouse embryo fibroblasts), Sirc (rabbit cornea fibroblasts), and Te-671 (human rhabdomyosarcoma). Values were estimated from Fig. 2 of Ref. [98] for HEK293A cells. ^g^ Readthrough efficiency was confirmed in a longer construct (−485 UGA +127). ^h^ “Fig.” refers to the figure in the original publication cited in the corresponding reference. ^i^ Relative to the no-element construct (=100%). ^j^ Readthrough activity supporting subgenomic replication was observed in Huh7 (human hepatocellular carcinoma), SVG-A (human astroglia), RD (human rhabdomyosarcoma), C2C12 (murine myoblast), and C6/36 cells. ^k^ Sf21 cell-free translation extract (Shimadzu, Japan).

The efficiency of readthrough is defined as the ratio of ribosomes directed to readthrough to those that reach the readthrough termination codon. Experimentally, efficiency is typically estimated using a positive control mRNA in which the stop codon at the end of ORF1 is replaced with an appropriate sense codon, allowing translation to continue to ORF2. Negative control mRNAs are sometimes difficult to establish because the stop codon context is often chosen for its intrinsic readthrough activity. Moreover, similar protein outputs can arise through distinct mechanisms, such as alternative splicing in related species. In practice, background levels can be assessed using an additional in-frame downstream stop codon, as recommended [103], although no single approach is definitive. Readthrough products can be measured either as an ORF1–ORF2 fusion protein [93] or via 2A peptides separating the ORF1 and ORF2 reporters [101]; in both cases, the efficiency, as described above, provides a comparative measure of translational fidelity.

Readthrough efficiency is influenced by multiple factors. First, the actual values are influenced by factors such as the use of cell-free systems or cultured cells, and the design of reporter constructs. Second, efficiency is modulated by *trans*-acting factors, such as viral polypeptides expressed during infection that increase readthrough [95,96], the exogenously expressed ribosomal protein uL4 that stimulates readthrough [98], or the interferon-induced protein Shiftless that inhibits readthrough [104,105], and by environmental or host cellular conditions, such as pH [97], temperature (28 °C or 37 °C) [85], or restrictive invertebrate host cellular conditions [102]. Under standard vertebrate cell conditions (i.e., in the absence of known added *trans*-acting factors and at approximately 37 °C), reported values range from 2% to 15% (Table 3). Readthrough efficiencies at bacterial UGA codons, in the absence of nonsense suppressor tRNAs, have been reported to be 3–5% in the Qβ coat protein of *Escherichia coli* [106] and 1–6% in the constitutively expressed *cat-86* gene of *Bacillus subtilis* [13]. These observations indicate that bacterial readthrough efficiency is influenced by both experimental conditions (as seen in Qβ) and codon context (as seen in *cat-84*), consistent with the variability in Table 3.

### 2.5. Functional Implications of Beier and Grimm Classification

The current classification is based on the functional significance of conserved nucleotides within ±3 codons from the readthrough termination codon and reflects their cross-species activity and the requirement for the downstream stimulatory structure [1].

Type I nonanucleotide UAG CAR YYA, first identified in TMV, functions across phylogenetically diverse eukaryotic systems, including plants [9,74], mammals [9,27,64,107,108], and yeast [109,110]. Its activity does not require a stimulatory downstream structure [3,74,110]. In plants [74] and yeast [111], this motif can also promote readthrough across all three stop codons.

In contrast, type III UAG GGR NGU CAG, studied in MoMLV, is weak even in the autologous mammalian assay system [75,107]. However, its activity is markedly increased by a downstream H-type pseudoknot starting from nucleotide +12 [61,62,63]. In plants, type III UAG GGA GGC in carnation Italian ringspot virus (CIRV) requires a long-distance RNA–RNA interaction spanning > 3.5 kilobases (kb) for readthrough [65]. Thus, the type III context most likely generally requires a downstream stimulatory structural element.

Type II includes animal virus UGA readthrough events, some of which overlap the UGACUA motif frequently identified in mammalian genes [27,57,112,113]. Based on subsequent findings, the mechanisms can be heterogeneous. This type comprises two different hexanucleotides: (i) UGACGG, present in both plant and animal viruses such as VEEV and CTFV, and (ii) UGACUA, present solely in animal viruses such as CHIKV, SINV, and PSIV. VEEV and CTFV require a downstream extended stem–loop, with readthrough efficiency reduced to less than 1% in its absence [64,66]. In contrast, a downstream stimulatory structure is conditionally required for UGACUA [64,67,68,69,72]. A derivative of the UGACUA motif, UGACUAG, conserved in 23 human genes, supports at least ~1.3% readthrough efficiency [112], suggesting that some UGACUA-related motifs possess intrinsic basal readthrough activity that varies depending on sequence context and experimental conditions. Notably, SINV readthrough efficiency in mosquito C6/36 cells is enhanced from 10% to 24% by the downstream structural elements [85], suggesting that the degree of stimulation is host-dependent. Since downstream structures may differ even within the same virus genus (see Section 4), the stimulatory impact of these elements must be evaluated individually for each virus. Thus, type II viral readthrough with the UGACUA motif may be regulated in a structure-dependent manner that varies among individual viruses, potentially in combination with additional host-dependent factors.

## 3. Viral Strategies to Compete with Canonical Termination

As stop codon readthrough is one type of recoding [6,60,70], signals encoded in mRNA modulate the kinetics of normal translation steps. In RNA virus frameshifting, another type of recoding for which the mechanistic understanding is relatively advanced, the translocation step of elongation can be disrupted by pauses induced by the nascent peptide and/or downstream RNA structures. In stop codon readthrough, where neither translation termination nor reading frame shifts occur, canonical termination is necessarily disturbed during decoding. In animal virus readthrough, where no cognate tRNAs (those matching the codon through standard codon–anticodon interactions) are available, this outcome is thought to arise from competition between near-cognate tRNA selection and termination pathways at the ribosomal A site, which biases the decoding pathway toward either continued elongation or peptide release (Figure 1A) [1,2,3,5,6]. The anticodon of these tRNAs can form noncanonical interactions with the termination codon [1]. Note that the term natural suppressor tRNAs [1,2,5] has been used to encompass both near-cognate and non-cognate decoding events.

Figure 3A summarizes a bacterial kinetic framework for translational decoding [114,115], extended here to eukaryotic elongation. Early bacterial pre-steady-state kinetic studies using fluorescently labeled tRNAs resolved tRNA selection into kinetically distinct phases and defined rejection pathways associated with kinetic proofreading steps (*k*_1_–*k*_7_) [114,115]. This framework was subsequently refined by structural studies [51] and extended by ensemble kinetic analyses [116,117,118].

Direct experimental assignment of readthrough effects to specific kinetic transitions remains lacking. Nevertheless, bacterial frameworks provide a useful basis for inferring stepwise processes of eukaryotic elongation and termination (Figure 3A,B), despite the limited availability of comprehensive kinetic measurements in eukaryotic translation. Within this context, Section 3 organizes current knowledge by considering individual stages of decoding and termination, highlighting mechanistic features that may contribute to translational readthrough.

### 3.1. Near-Cognate vs. Cognate Elongation

Translation mediated by near-cognate tRNAs has been analyzed as a translational error during elongation [56]. To achieve this level of translational accuracy, near-cognate tRNAs are rejected during successive steps of elongation [51,52,115,116,117,118,119,127,128,129,130,131,132] (Figure 3A). The minimal set of defined checkpoints consists of initial selection and kinetic proofreading. Near-cognate decoding at sense codons has been extensively characterized in *Escherichia coli* (*E. coli*), *Thermus thermophilus* (*T. thermophilus*), and human ribosomes. All of these experiments support the rejection of near-cognate tRNAs during initial selection. The values of the dissociation rate constant *k*_−2_ are affected by codons and Mg^2+^ concentration. Under high-fidelity (HiFi) conditions (3.5 mM MgCl_2_), the *k*_2_*/k*_−2_ discrimination factor has been reported to range from 348 to 2000 [116,117,118].

Structurally, during initial selection, the 18S rRNA nucleotides A1824 and A1825 in helix h44, together with G626 in h18 (corresponding to A1492, A1493, and G530 in *E. coli*), recognize the minor groove of the RNA helix adopting Watson–Crick geometry [119,121,133] (Figure 3C), an interaction that near-cognate tRNA cannot support, except in cases where a rare tautomer (a transient alternative form of a nucleobase) adopts a Watson–Crick-like geometry (for bacterial examples, see [134,135,136,137,138,139,140,141]). This A–minor interaction is believed to provide a kinetic advantage that allows cognate tRNA to progress through the downstream steps of GTPase activation (*k*_3_), GTP hydrolysis (*k_GTP_*), and kinetic proofreading, ultimately enabling the transfer of the peptidyl group to the aminoacyl tRNA (*k_pep_*).

Kinetic proofreading in the ribosome constitutes the second selection step in decoding and uses the free energy from GTP hydrolysis to selectively reject mismatch-containing tRNAs. This energy-dependent mechanism has been proposed for high-fidelity enzymatic reactions such as DNA replication and tRNA aminoacylation [142,143]. Based on kinetic studies in *E. coli* [115,116,117,118,144], initial selection strongly discriminates between cognate and near-cognate tRNAs (*k*_2_*/k*_−2_: 348-fold difference; [116]), whereas kinetic proofreading provides a weaker, yet still significant, additional enhancement of translational fidelity (proofreading factor = *k*_5_/(*k*_5_ *+ k*_7_): 17-fold; [116]) by further rejecting near-cognate tRNAs. Proofreading is likely composed of two consecutive phases [144,145]: factor rearrangement and accommodation, during which the aminoacyl-tRNA fully enters the A site after initial codon recognition (Figure 3A). While proofreading efficiency may arise from kinetically distinct contributions from these two phases, this review uses the effective proofreading factor derived from competing rate constants because it provides a widely used and experimentally comparable measure.

Elongation proceeds through multiple states, as originally described in the framework (Figure 3A), and has been increasingly resolved into more detailed structural intermediates in both eukaryotes [119,146,147,148,149] and bacteria [150,151,152], exhibiting distinct kinetics [119], as well as tRNA conformational changes [149,153] and dynamic movements during accommodation [149], thereby reflecting differences in the host translational machinery. Ribosomes undergo continuous conformational changes during these transitions, which are accompanied by tRNA conformational changes, as observed in bacteria [154,155,156,157]. The Watson–Crick geometry between the tRNA anticodon and mRNA codon ensures efficient translation by providing sufficient structural space for conformational changes and thermodynamic stability during ribosome dynamics.

GTP hydrolysis of decoding-related translational GTPases (EF-Tu and eEF1A in elongation) provides the energy for subsequent rearrangements of decoding components (tRNA accommodation in elongation) and the second-round selection (kinetic proofreading), ultimately leading to the release of inorganic phosphate (Pi) and GDP-bound GTPase from the ribosome (Figure 3A). The reaction occurs at the GTPase-associated center (GAC), composed of the sarcin–ricin loop (SRL) and the P-stalk (bacterial L7/L12 stalk) of the large subunit (LSU), and requires GTPase activation, in which the catalytic histidine residue moves toward a conserved adenylate in the SRL (A4607 in mammals; A2662 in *E. coli*). During cognate elongation, GTPase activation is associated with closure of the small-subunit (SSU) shoulder domain toward the SSU body in bacteria [137,157,158] or with a corresponding conformational rearrangement in mammals [121,133]; these movements are collectively termed domain closure in this review. Domain closure is induced by A–minor interactions at the decoding center and is thought to couple translational fidelity with the efficiency of canonical translation.

#### 3.1.1. Ribosomal Mutations Affecting Fidelity During Near-Cognate Elongation

In bacteria, near-cognate elongation (i.e., translational errors) can influence growth in the presence of aminoglycosides, such as streptomycin or paromomycin. Early genetic studies by Gorini and colleagues distinguished streptomycin-resistant or “restrictive” (hyperaccurate) mutations (*str*; now termed *res*) from ribosomal ambiguity (*ram*) mutations that increased translational errors [159,160]. These mutations are located at distinct interfaces involved in the domain closure of SSU ribosomal proteins, including uS12, uS4, and uS5. Specifically, a uS12 *res* mutation (e.g., Lys42Asn [132]) is located at the uS12/h27/h44 interface near the decoding center and has been shown to increase cognate selectivity during proofreading (further reducing *k*_5_/(*k*_5_ *+ k*_7_) for near-cognate tRNAs), thereby limiting the completion of near-cognate elongation after GTP hydrolysis. In contrast, a uS4 *ram* mutation at the uS4–uS5 interface (e.g., a five-nucleotide deletion, C528–A532; resulting in a C-terminally truncated protein [132]) decreases the *k_−_*_2_ of near-cognate tRNAs during the initial selection stage, allowing more near-cognate tRNAs to proceed to GTPase activation and resulting in higher translational error rates even in the absence of aminoglycosides [130,132,161].

Translational fidelity mutations arising from corresponding ribosomal proteins have been observed in both yeast and the filamentous fungus *Podospora anserina*. In yeast, these include accuracy alleles in uS12 and omnipotent suppressor alleles (*sup46* and *sup44*) in uS4 and uS5 [162,163,164], whereas in *Podospora*, mutations have been reported in S7/S9 (uS4), S1 (uS5), and S19 (uS12) [165], with the shorter S7 form resembling the conserved uS4 in yeast RPS9A and the canonical human (MANE) isoform. Notably, *Podospora* accuracy mutations derived from S7 (190 aa, with a slightly lower isoelectric point (pI)) and S9 (220 aa, with a slightly higher pI) are accompanied by shifts in protein pIs, which in turn are associated with either increased or decreased translational fidelity [166,167]. This finding is consistent with the earlier observation in yeast *sup46* alleles [168], where uS4 (previously referred to as S11 in some yeast-based annotation systems; see [169] for variations in nomenclature) exhibits allele-dependent changes in pI. These findings suggest that electrostatic interactions at the uS4–uS5 interface are likely to contribute to the ribosomal rearrangements required for GTPase activation during near-cognate elongation, which is consistent with recent genetic studies in yeast [170].

The presence of analogous mechanisms across kingdoms, together with an isoform with a different pI in *Podospora*, suggests that rates of near-cognate elongation can increase under conditions in which altered translation is advantageous. Indeed, increases in translational errors can be beneficial under stress, potentially supporting adaptation [171,172,173], whereas excessive reductions in translational fidelity can be detrimental in higher eukaryotes [174]. Subsequent studies using genetically engineered mice carrying ribosomal fidelity mutations have further linked reduced ribosomal fidelity to aging-associated phenotypes [175,176], supporting the physiological importance of maintaining decoding accuracy in higher eukaryotes. Collectively, these observations highlight the importance of understanding how structural and kinetic constraints shape the fidelity landscape of decoding.

#### 3.1.2. 16s rRNA ram Mutations

In bacteria, several missense suppressor mutations, whose phenotypes resemble *ram* mutations, have been isolated in the 16S rRNA gene at positions both near and outside the uS4–uS5 interface [177]. Rates of ribosome-stimulated GTP hydrolysis are elevated for both cognate and near-cognate tRNAs [130,178,179]. Because near-cognate rejection during kinetic proofreading is also relaxed [130], a subset of these suppressors may constitute a distinct class of 16S rRNA-derived *ram* mutations, differing from those isolated in the uS4 and possibly uS5 genes [132]. Consistently, structural analysis of representative 16S *ram* mutations G299A (equivalent to human G419A) and h14 G347U (for which no clear human equivalent has been established) reveals conformational changes extending beyond the immediate mutation sites. Whereas G347U perturbs h14 within the intersubunit bridge B8 [178], both G299A and G347U display inward movements of the SSU shoulder, even in the absence of the A-site anticodon stem–loop (ASL) [179], suggesting that *ram* phenotypes can arise through long-range structural communication pathways extending beyond the uS4–uS5 interface.

Eukaryotic *ram*-like mechanisms, identified thus far mainly through mutations affecting the uS4–uS5 interface, may similarly arise through long-range structural communication pathways that influence decoding, although the underlying structural basis remains unclear. In contrast, GTPase activation during termination, which does not require A–minor interactions or domain closure [121], likely differs from that in elongation—including near-cognate elongation—due to fundamentally distinct decoding center geometries during codon recognition (Figure 3C,D).

### 3.2. Normal Termination and Its Cis-Elements

Under normal conditions, stop codons are typically recognized efficiently by release factors, leading to the termination of translation and release of the nascent polypeptide. Peptide release in eukaryotes is a late step in the termination process, occurring after eRF3 GTP hydrolysis and eRF1 accommodation at the peptidyl transferase center [120,180] (Figure 3B modeled on elongation in Figure 3A). Following eRF3–GDP dissociation, ABCE1 (ATP-binding cassette subfamily E member 1) associates with the post-termination complex and promotes ribosome recycling through ATP-driven ribosomal subunit dissociation, leading to the release of eRF1 and ABCE1 [181,182]. The following subsections describe the proposed progression of normal termination [120,183], drawing mainly on mammalian evidence while considering genetic studies in yeast, and introduce *cis*-acting elements that may influence readthrough.

#### 3.2.1. Termination Codon Recognition

In eukaryotes, all three termination codons are recognized by a single class I release factor, eRF1. The nucleotide at the fourth position (+4) influences termination efficiency, as it does in bacteria. Purines at the +4 position—more frequently found in highly expressed eukaryotic genes that require efficient translation termination [184]—biochemically promote more efficient peptide release in reconstituted mammalian peptide release assays [185,186]. In contrast, cytidine at the +4 position increases readthrough efficiency in all three codons in yeast [187].

Addition of eRF1 alone in the reconstitution system induces the downstream movement of terminating ribosomes by two nucleotides (toeprint assay [188,189]) or conformational rearrangement of 80S ribosomes, primarily affecting 28S rRNA (chemical footprint [190]). Footprinted sequences in 18S rRNA within h33 and in the h34–h35 loop likely reflect head rearrangements, whereas those in the h18 G626 loop point to the involvement of the decoding center in codon recognition (see below). Taken together, these observations from the reconstitution system suggest that during termination codon recognition, 80S ribosomes undergo a conformational change involving the LSU, in contrast to elongating ribosomes, which undergo domain closure.

Structurally, the eRF1 N domain (142 aa in mammals) enters the A site and plays a pivotal role in discriminating termination codons from cognate sense codons at the ribosomal A site [121,191,192], confirming the results of previous biochemical and genetic studies [193,194,195,196,197]. The +1U nucleotide, common to all three termination codons, is recognized by the eRF1 N domain TAS-N_61_IK_63_S motif [123,191,192,198]. The +2 and +3 purines (except for UGG) are recognized by the hydrogen-bonding network formed by two conserved eRF1 motifs and Glu55 [188,196,199,200,201,202]. The resulting cognate interaction between the mRNA termination codon and eRF1 (decoding factor) is distinct from that during elongation in both orientation and spacing within the A site (Figure 3C,D). Additionally, compared with an elongator tRNA, the eRF1 N domain occupies a more head-proximal region adjacent to 18S rRNA helices h30–h31. Together, these observations indicate that termination codon recognition requires a ribosomal interaction mode distinct from that used during elongation. Notably, the positioning of the eRF1 N domain appears to be unique even among currently characterized eukaryotic A-site-occupying proteins. For example, Pelota (Dom34 in yeast) [121,203] and angiogenin [204] engage the decoding center through distinct binding geometries and functional contexts, highlighting the specialized architecture of eRF1 required for stop-codon recognition.

Consistent with this notion, cryo-EM structures reveal a compact mRNA arrangement, accompanied by rRNA-mediated stacking interactions that are not observed during elongation. Across all four ribosomally recognized termination codons examined by cryo-EM—including three termination codons followed by +4G [191] and an engineered UAAAaa sequence in the human cytomegalovirus (HCMV) gp48 transcript [192] (A-site nucleotides capitalized)—the adjacent +4 nucleotide is positioned in the A site and stabilized by stacking with the monitoring nucleotide G626 of h18. The +2 purine at the termination codon forms stacking interactions with both the flipped-out h44 A1825 and the +3 purine, contributing to mRNA compaction. Additionally, in the case of the UGAGcu sequence analyzed in rabbit 80S [121], the downstream +5 nucleotide is stabilized by base stacking with C1698 (C1397 in *E. coli*) at the mRNA entry channel. Thus, terminating ribosomes with eRF1 bound at the A site recognize and stabilize a region of up to five nucleotides that includes the termination codon through base-stacking interactions.

Quantitative studies indicate that purine–purine interactions often exhibit more favorable stacking energies than pyrimidine–pyrimidine interactions, reflecting the larger π-electron surface and greater polarizability of purines [205]. Although these measurements were performed with DNA, the trend that purine rings provide greater stacking free energy than pyrimidine rings is also expected to hold in RNA. Bacterial 70S and RF2 crystal structures have shown that 16S h18 G530 (18S G626 in humans) interacts with the +3 adenine of the UAA [206] or UGA [207] termination codons through stacking interactions. The latter study proposed that the difference in stacking free energy between purine–purine and purine–pyrimidine bases contributes to discrimination between UGA termination codons and UGY sense codons, with Y denoting pyrimidines (C and U). Together with preliminary biochemical observations [186] that UGACR may promote more efficient peptide release than UGACY, certain pentanucleotides containing a +4Y/5Y motif may influence codon recognition efficiency in eukaryotic systems, consistent with the possibility that downstream stacking interactions contribute to termination efficiency. In a reconstituted system containing *Artemia salina* (brine shrimp) ribosomes, human eRFs, and patient-derived PTCs as mRNA templates, the apparent affinity constant (*K_A_*), derived from Hill equation fitting, showed increased values under +5Y termination contexts, indicating reduced binding efficiency in these contexts [208]. Notably, certain termination contexts, including those containing the UGACUA sequence, exhibited sigmoidal rather than hyperbolic binding behavior, suggesting that downstream sequence context may influence not only apparent affinity, but also the kinetic pathway of release factor engagement.

Conversely, unless +5A is followed by the sequence mimicking the type I readthrough motif (AAUUA from +5), the presence of a single purine at this site is sufficient for efficient termination [109].

#### 3.2.2. eRF3 GTPase Activation

Termination GTPases differ across domains: RF3 functions post-release in bacteria, whereas archaea use aEF1 (elongation factor) as a termination GTPase. The trimeric complexes aRF1–aEF1–GTP (archaea) and eRF1–eRF3–GTP (eukaryotes) bind terminating ribosomes before peptide release [123,209,210] (Figure 3B).

GTPase activity on eRF3 is strongly ribosome-dependent and also requires eRF1, as originally demonstrated by Frolova et al. [211] and further characterized by detailed molecular and kinetic analyses [182,212,213]. Ribosomal GAC, composed of the SRL and P-stalk in the LSU, is involved in eRF3-dependent GTP hydrolysis [48,214,215]. The C3025U mutation in 25S rRNA—equivalent to SRL 4603 in humans and 2658 in *E. coli*—increases stop codon readthrough in yeast, illustrating the functional relevance of SRL. Chemical footprinting and structural analyses revealed interactions between the C-terminus of the eRF1 C domain and the base of the P-stalk, comprising uL11 and 28S rRNA helices H43 and H44 [121,123,190]. Supporting this view, mutation alleles affecting the eRF1 C-terminal end [216], as well as the depletion of uL11 [48], increase translational readthrough, highlighting the active involvement of these elements in translation termination. These observations further suggest that the eRF1–P-stalk interface may contribute to the positioning of eRF3 and facilitate GTPase activation, although direct mechanistic evidence remains to be established.

Structurally, ribosome domain closure induced by decoding nucleotides, which ensures rapid navigation of eEF1A catalytic His95 in the G domain close to the SRL, is not observed in terminating ribosomes [121]. The observed absence of domain closure is likely attributable to the lack of A–minor interactions, especially the apparent absence of the flipped-out conformation of h44 A1824 during termination, accompanied by an altered trajectory and stabilization of the A-site codon compared with elongation (Figure 3C,D). The alternative mechanism by which the catalytic His residue (His300 in human eRF3A) is activated remains unclear, partly because structural information on its position prior to activation is lacking.

#### 3.2.3. Post-GTP Hydrolysis State During Termination

Upon GTP hydrolysis by eRF3, the ribosomal complex undergoes rearrangement of the eRF1–eRF3–GDP complex and accommodation of the eRF1 M domain, enabling extrusion of the conserved GGQ motif into the LSU peptidyl transferase center (Figure 3B). Given the stable occupancy of the eRF1 N domain at the termination codon in the ribosomal A site [121,192], a kinetic proofreading mechanism operating during these rearrangements has been proposed, in which the free energy derived from GTP hydrolysis destabilizes eRF1 interactions with near-cognate sense codons such as UGG [217], thereby contributing to termination fidelity [120].

Productive accommodation is followed by peptidyl-tRNA hydrolysis, committing the ribosome to termination and preventing further elongation via readthrough. Accordingly, factors influencing readthrough are expected to exert their primary effects before accommodation is completed. The following subsections discuss additional factors influencing termination, with a particular focus on their relevance to viral readthrough, including ribosome recycling and the role of 3′ UTR length.

#### 3.2.4. Role of Ribosome Recycling

Eukaryotic translation termination is tightly coupled to ribosome recycling and the subsequent round(s) of initiation, including reinitiation [120,180] as supported by a range of experimental approaches [218,219,220,221,222]. Depletion of ABCE1 (Rli1 in yeast) increases readthrough in both mammals and yeasts [223,224], consistent with Rli1 overexpression in yeast [225], indicating that slowing termination allows more frequent near-cognate elongation. To facilitate ribosome recycling and subsequent events, it has been proposed that in some mRNAs from multicellular animals, mRNA–rRNA complementarity at the 3′ UTR exists between the first fifty nucleotides of the mRNA 3′ UTR flanking the termination codon and the 18S rRNA near the mRNA entry pore [226]. Given this complementarity, the post-termination ribosomal complex has been suggested to move from the termination site toward ribosome recycling. Although direct biochemical evaluation of this sequence in translation, including viral translation, has not been obtained, the proposal raises the question of how long ribosomes can remain at the termination site. If ribosomes accumulate excessively and trigger ribosome-associated quality control (RQC), thereby leading to template RNA degradation, this would be detrimental to viral replication.

Ribosome accumulation at the termination codon can be assessed by ribosome profiling. Accurate analysis in yeast revealed the accumulation of footprints derived from terminating and/or recycling ribosomes 15 nucleotides upstream of the UAA termination codon, corresponding to the 5′ end of ribosome-protected footprints [227]. Early ribosome profiling studies in mammalian cells, including those by Ingolia, Weissman, and colleagues [228,229], analyzed ribosome occupancy on individual genes and ORFs, revealing heterogeneous ribosome behavior around start and stop codons. Ribosome profiling analyses of viral translation in a gammaretrovirus (Figure 1 in Ref. [100]) and a plant ilarvirus (Figure 5 in Ref. [230]) revealed relatively limited ribosome accumulation at readthrough sites, even though the number of investigated viruses remained small. Recent advances in sequencing-based approaches that selectively capture termination-associated ribosomes [231] may enable more detailed investigations of ribosome behavior at readthrough sites, providing a framework to assess potential delayed termination under these conditions.

#### 3.2.5. Cytoplasmic Poly(A)-Binding Protein (PABPC1) Involvement and Effects of 3**′** UTR Length

The termination GTPase eRF3 (637 aa in human eRF3A) contains an extended N-terminal region (206 aa) that varies across eukaryotes and may contribute to species-specific regulation of translation termination. In mammals, this region harbors PABP-interacting motif 2 (PAM2), which mediates interaction with PABPC1 via its C-terminal MLLE domain [232,233,234].

PABPC1–eRF3 interactions play a pivotal role in translation termination, as disruption of this interaction reduces cap-dependent translation in both a cell-free system and HeLa cells [235], and impairs eRF recruitment and the formation of the downstream termination complex that recognizes the stop codon in a reconstituted system [236]. Consistent with a role for PABPC1 bound to the poly(A) tail in the 3′ UTR, readthrough at the UGACAA site increases with increasing 3′ UTR length in yeast mutants lacking Pab1 (yeast PABPC1 [237]).

Genome-wide ribosome profiling identified human cellular readthrough genes in HEK293T cells, and a machine-learning analysis further showed that shorter 3′ UTR length (approximately 100–1000 nt) is one of the nine *cis*-elements that reduce readthrough efficiency [238]. This effect is likely mediated by PABPC1, which binds the poly(A) tail via its RRM1–2 domain and interacts with eRF3.

Because viral readthrough sites are typically followed by long coding sequences (often ≥1000 nt; Table 1), the PABPC1-dependent reduction in readthrough is expected to be minimal in most viral cases. Nonetheless, cellular termination remains a strong competitor, as eRF1–eRF3 can mediate peptide release with distinct kinetic parameters, even in the absence of PABP, as shown using a domain-deleted eRF3 mutant [239].

### 3.3. Cis-Acting RNA Elements Promoting Viral Readthrough

Recent advances in recoding and eukaryotic cellular gene readthrough have highlighted the need to update our understanding of viral readthrough *cis*-elements. In this section, I discuss these elements in order of their location from the 5′ end. Although the full mechanistic landscape of eukaryotic termination remains incomplete—as illustrated by eRF3 GTPase activation—potential molecular targets for readthrough, particularly in relation to the two selection stages described in Section 3.1 (namely, stop codon recognition and near-cognate elongation during kinetic proofreading), are highlighted in the relevant subsection.

#### 3.3.1. Stop Codon Context—A Historical and Methodological Perspective

Early bacterial readthrough studies using suppressor tRNAs clearly demonstrated that flanking sequences around the termination codon strongly influence readthrough efficiency [240,241,242]. These effects were described by various terms, including “reading context” [243], “adjacent effects” [244], and “mRNA context” [241]. It is therefore unsurprising that similar influences were already considered or later identified in viral readthrough and likewise referred to as context [9,62,84,90]. The precise range and definition of “context” have changed over time, varying among researchers, and sometimes even extending to the very end of the mRNA. Now that atomic-level information on the mRNA path through the ribosome is available—and given that validated frameshifting stimulatory structures act from outside the ribosome—the intra- and extra-ribosomal influences on readthrough can be more precisely distinguished. In this review, I refer to these as “stop codon context” for the immediate codon-proximal region and “downstream structure” for elements acting outside the ribosome.

While the signals for recoding should ideally be understood along a single, continuous mRNA molecule, the knowledge accumulated over decades has rendered it increasingly difficult to discuss them in a strictly linear fashion. Separate discussions by functional regions became inevitable, allowing the complex information to be organized more clearly without losing sight of the underlying molecular mechanisms.

*(1)*  *Upstream Elements*

Effects of upstream elements have not been intensively studied in animal virus readthrough. Mechanistically, the effects can be divided into two: (i) amino acid sequences encoded in an upstream element, and (ii) nucleotide sequences flanking the readthrough termination codon.

(i) *Upstream Amino Acid Sequence:* In eukaryotes, upstream coding sequences can arrest termination, from AUG-stop elements in plants [245] to short upstream open reading frames (uORFs) in viruses and fungi [246,247]. This phenomenon is distinct from stop codon readthrough, which permits productive elongation beyond the termination codon. In the HCMV gp48 transcript, the arrest peptide of uORF2 engages the ribosomal exit tunnel and prevents peptidyl-tRNA hydrolysis, even though eRF1 is fully accommodated and recognizes the UAA termination codon [192]. Thus, termination is kinetically trapped during eRF1 accommodation (uORFs) or by premature accommodation (AUG-Stop). The resulting ribosome arrest can engage quality-control pathways, including mRNA degradation in fungi and plants; however, under nutrient starvation, downstream translation may become permissive in fungi and plants. Importantly, these events do not involve changes in genetic information. Rather, they illustrate that kinetic traps can broadly contribute to translational control without necessarily involving recoding.

Effects of nascent amino acid sequences on cellular readthrough have recently been analyzed using machine-learning approaches. Notably, no significant contribution from the nascent peptide sequence to stop codon readthrough was detected, except for the P-site amino acid, which may be mediated by the corresponding tRNA (Figure 1b and Figure 2d in Ref. [238]). Thus, there is currently no evidence that the interactions between the nascent peptide and the ribosomal exit tunnel influence stop codon readthrough. Nevertheless, it remains conceivable that the nascent peptide influences an earlier step in decoding. Although the timing and context differ, kinetic regulation from upstream coding sequences has been identified in viral frameshift events. In the SINV structural gene [248,249], this involves the co-translational folding of the E2 transmembrane region, whereas in the severe acute respiratory syndrome coronavirus 2 (SARS-CoV-2) NSP11/12 gene [125], it is mediated by the NSP10 nascent peptide.

(ii) *Upstream Nucleotide Sequence:* In several eukaryotic readthrough contexts, the nucleotide sequence immediately upstream of the stop codon has been implicated in modulating readthrough. For example, in the human opioid related nociceptin receptor 1 (OPRL1) gene, a P-site codon upstream of the UGACUAGGC motif contributes to readthrough efficiency [250]. Early studies examining CAN and GGN as preceding codons suggested a preference for an adenylate at the −1 position upstream of UAG stop codons [251]; however, broader analyses across extended codon species or transcriptome-wide datasets indicate that such a preference is not absolute [238,250,252]. The underlying determinants may include specific nucleotides, encoded amino acids, or properties of the corresponding P-site tRNA, with their relative contributions remaining difficult to disentangle experimentally across currently sampled codon contexts [250]. Machine-learning analyses suggest that readthrough modulation cannot be explained solely by specific amino acid or nucleotide identities, raising the possibility that properties of the corresponding tRNAs may contribute more generally to readthrough regulation [238]. Recent fluorescence-based assays of readthrough in malate dehydrogenase-1 (MDH1) constructs containing the UGACUAGAC motif show modulation of readthrough efficiency by simultaneous mutations at A−9 and A−8 [252]. These effects are difficult to attribute solely to either nucleotide identity or amino acid differences, as also suggested for P-site codon effects [238,250].

Previous studies collectively suggest that upstream nucleotide context extending to the −3 position, rather than only the nucleotide at the −1 position, contributes to the efficiency of stop codon suppression or readthrough. In *Salmonella typhimurium*, including upstream nucleotides, up to −3 improves the prediction accuracy of suppression efficiency in *LacI–Z* fusion constructs (Figure 1 in Ref. [240]). Similarly, among human genes sharing the UGACUAG heptanucleotide motif, readthrough efficiencies were more comparable when the upstream context up to −3 was considered (Figure 1 and Table 1 in Ref. [112]). Notably, pairs of genes with identical −3 to −1 sequences exhibited nearly identical readthrough efficiencies, whereas the remaining differences were small relative to the overall dynamic range.

Despite these observations, viral readthrough assays have frequently employed native upstream viral coding sequences without systematic mutational or mechanistic analyses (Table 1 and Table 3), leaving the contribution of nucleotides at positions −3 to −1 largely unexplored.

*(2)*  *Stop Codon Identity*

Effects of replacing the viral readthrough termination codon with the other two codons have been investigated in several viral sequences. In type II readthrough for SINV, CHIKV, CTFV, and PSIV, the UGA codon supports high readthrough efficiency. Three independent translation-based assays, either including downstream RNA structure elements [66,67] or lacking these elements [72], showed only limited readthrough at UAG or UAA codons, whereas two studies [69,85] reported that the UAG mutant shows intermediate apparent activity (~20–33% of UGA), while UAA remains at background levels. In type III MoMLV, wild-type UAG and UGA exhibit comparative activity, whereas UAA substitution reduces readthrough efficiency to approximately 35–40% of wild-type levels [99]. Despite higher readthrough at UGA, viral replication is typically more efficient with UAG, as reflected by independent viral titer measurements [83,91,99]. Regarding readthrough in this type, the presence of the stimulatory downstream structure is crucial [61,62,63,75,99].

Collectively, these observations indicate that UGA is a versatile and highly efficient codon for readthrough in mammalian systems. Beyond type II stop codon contexts, UGA can also support type III MoMLV readthrough [99] and substitute for type I TMV UAGCAAUUA elements in a homemade RRL [75]. Note that this versatility can be host-dependent; in plant cells, the type I TMV context prefers UAG over UGA [74].

UAG in mammals supports measurable readthrough, providing efficiencies of 2.5–12% for MoMLV readthrough (Table 3). The marked inefficiency of UAG in all type II sequences tested highlights the importance of the stop codon context. Thus, the UAG sequence may not fully meet the requirements imposed by this context.

UAA is consistently a weak readthrough signal in near-cognate elongation, including assays in reconstituted mammalian systems [253]. UAA readthrough has been observed in reconstituted systems combining *Artemia salina* ribosomes with heterologous release and elongation factors [208], although the experimental design and calculation of readthrough efficiency differ from those used in conventional reporter-based readthrough assays discussed in this review.

Ribosome profiling in mammals has revealed low-frequency readthrough at all three termination codons [25,57], indicating that initial selection alone does not fully eliminate near-cognate elongation. Observed efficiencies of ~10% at viral readthrough sites suggest the involvement of kinetic proofreading in near-cognate selection. This host proofreading mechanism may contribute to shaping readthrough efficiency. The following subsections outline near-cognate tRNAs in mammalian cells, including the contributions of kinetic proofreading and host RNA degradation, notably nonsense-mediated mRNA decay (NMD).

(i) Near-cognate tRNAs and Kinetic Proofreading

Evidence that near-cognate tRNAs decode termination codons comes from analyses of the amino acids incorporated at UAG and UGA codons [46,47,92,111,254,255,256,257,258,259,260], with UAG readthrough most frequently resulting in Gln, Tyr, or Lys and UGA readthrough most frequently yielding Arg, Cys, or Trp. Consistently, exogenous expression of selected near-cognate tRNA species enhances readthrough [261,262,263]. Note that the UAG codon in the MoMLV mRNA sequence is exclusively decoded as Gln [46,92]. In contrast, the rabbit β-globin UGA site (hexanucleotide UGAGUA) exhibits highly diverse decoding outcomes in rabbit reticulocytes, allowing the incorporation of Arg, Cys, Ser, or Trp, as well as cases with no amino acid incorporation [254]. Thus, the choice between UAG and UGA likely reflects a viral strategy to fine-tune readthrough, incorporating the identity of the amino acid at the termination codon, the downstream protein context, host tRNA availability, and, when present, the nucleotide needs of other *cis*-acting viral RNA elements.

Regarding near-cognate tRNAs that decode the UAG or UGA termination codons, several lack one of the two canonical Watson–Crick base pairs at the first or second codon positions, thereby perturbing the Watson–Crick geometry required for optimal A–minor interaction. Even in cases where canonical Watson–Crick base pairing can be maintained at these two positions, as observed for tRNA^Cys^(GCA), tRNA^Trp^(CCA), and tRNA^Tyr^(GUA), the overall decoding geometry is likely modulated by sequence context at the +3 codon position.

Single-molecule studies using *Artemia salina* ribosomes revealed unexpectedly frequent sampling events by tRNA^Trp^(CCA) at a UGA stop codon in a near-cognate context [128], suggesting that substantial interrogation of decoding geometry may occur during the initial selection. Consistent with these dynamic observations, ensemble cryo-EM analyses have shown multiple conformational states of the decoding center, including G530 (G626 in humans) conformational heterogeneity, supporting a structurally dynamic landscape during near-cognate decoding [137]. Single-molecule studies, using the UGACUA sequence, further revealed that approximately 20% of near-cognate tRNA trajectories reached an accommodated state within the observation duration, indicating that a substantial fraction of near-cognate decoding attempts can progress through kinetic proofreading to accommodation and that downstream sequence context may influence the progression of near-cognate decoding [128]. Another possible explanation is that the central G–C Watson–Crick pair formed by tRNA^Trp^(CCA) at UGA may provide additional stabilization of the decoding complex, thereby facilitating progression through kinetic proofreading despite the mismatch at the +3 codon position. This contrasts with earlier single-molecule studies, in which the mismatch involving tRNA^Phe^(GAA) was located at the central position of the UCU codon [119].

Experimental evidence from diverse systems, including genetic analyses, has implicated tRNA modifications in translational readthrough [264,265,266,267]. Whether similar modification-dependent mechanisms operate at animal viral termination codons remains unresolved. Notably, guide-directed pseudouridylation of decoding elements, including the +1 uridine of the stop codon and residues within the tRNA anticodon loop, has been shown experimentally to enhance stop codon readthrough [268,269,270], suggesting that chemical alteration of the codon and anticodon itself can modulate decoding outcomes. As our knowledge of mammalian tRNA modifications continues to expand [271,272,273,274,275,276], one or more modifications at positions 34–37, including the anticodon (positions 34–36), have been identified in most tRNAs involved in decoding readthrough codons, including Lys, Tyr, Arg, Ser, and Trp. tRNA^Gln^(CUG) likely remains a notable exception, based on classical depictions [108]. Although these modifications could, in principle, influence the conformational landscape of the anticodon–codon interaction during near-cognate decoding, current models of initial selection emphasize Watson–Crick geometry at the +1 and +2 codon positions. How tRNA modifications associated with stop codon readthrough contribute to near-cognate recognition in the ternary complex remains to be directly explored, partly due to technical challenges in incorporating modified tRNAs into reconstituted systems. Such modifications may play a role at later stages, such as kinetic proofreading, consistent with increased translational errors associated with *E. coli* near-cognate tRNAs possessing the 2-methylthio-*N*^6^-isopentenyladenosine (ms^2^i^6^A) modification at position 37 [277], including tRNA^Leu^(CAA) on poly(U) [278] and *trpT*(Su9) in UGA readthrough [279]. The contribution of tRNA modifications to decoding appears to differ across successive decoding stages. State-dependent free-energy calculations based on molecular dynamics showed that ms^2^i^6^A37 modification in tRNAs is associated with an increased energetic penalty of approximately 2.7 kcal mol^−1^ for near-cognate decoding in the A/T tRNA state [136], suggesting that the effects of this modification are stage-dependent, contributing to near-cognate discrimination at a pre-accommodation stage.

(ii) UAA (ochre) Mutations, NMD, and Viral Replication

Across all four genera of animal viruses examined, UAG or UGA—but not UAA—is consistently employed at the readthrough site, suggesting functional constraints on stop codon selection. Suppression of the UAA (ochre) codon by near-cognate tRNAs is generally weak and often undetectable in translation or replicon assays [15,29,66,67,68,69,72], consistent with effective kinetic proofreading, and can be observed in vitro when additional cellular tRNAs, including those from NIH3T3 cells, are supplied [91,92]. In one study, the readthrough efficiency in Vero cells was reported to be 2%, but the corresponding products were not readily detectable by immunoblotting [85].

Destabilization of mRNAs by NMD could further reduce ORF2 expression, as reported for several spliced transcripts [268,280]. Given that termination codons act as *cis*-elements involved in both readthrough and NMD, their role in RNA virus replication warrants further investigation, particularly in light of the dynamic interactions between viral RNAs and the host NMD pathway [281,282,283]. Importantly, animal virus readthrough, including retroviral *gag–pol* mRNAs, occurs on unspliced transcripts. Consequently, the contribution of NMD to these RNAs has yet to be fully elucidated, particularly in exon junction complex (EJC)-independent versus EJC-enhanced contexts [284,285,286].

Although the introduction of an ochre (UAA) codon clearly compromises readthrough translation, its effects on viral replication appear to vary among viruses and experimental systems. In several studies, UAA-containing mutants do not appear to be particularly detrimental to viral replication in virological assays [84,91,99,287], suggesting that near-cognate translational products may often suffice for viral replication. In contrast, other reports describe markedly reduced viral titers [68,83] or reversion events [82] associated with UAA codons. Viral recoding strategies in animal viruses appear to preferentially exploit termination codons that form relatively stable near-cognate interactions, thereby allowing the production of more downstream translational products. The possible involvement of host cellular countermeasures targeting this site, including ribosomal kinetic proofreading and NMD-mediated viral RNA degradation, remains to be fully clarified.

*(3)*  *Downstream Elements in the Stop Codon Context*

As the termination codon occupies the ribosomal A site, several downstream nucleotides traverse the mRNA entry channel. Structural studies suggest continuous mRNA density extending beyond the immediate stop codon and through the mRNA entry channel, reaching positions as distal as +11 or +12 from the stop codon in some complexes (Figure 3E). Recent functional assays of mammalian cellular readthrough suggest that nucleotides downstream of the hexanucleotide sequence used in the Beier–Grimm classification can modulate readthrough efficiency, potentially extending toward the mRNA entry channel region. For example, +8C increases readthrough in COS-7 cells, +9A in HEK293T cells, and +11G and +12C within the UGACUAG context in HeLa cells [238,252,288]. Because six-nucleotide sequences following the termination codon, derived from the TMV motif (CAAUUA), function in mammals (Section 2.5), sequence-level convergence does not appear to be unique, as further supported by the systematic saturation mutagenesis analyses [288].

Consistently, multiple downstream sequences derived from the CAAUUA type I TMV motif, as well as those derived from the CUAGGC motif, which overlaps with the type II motif, support readthrough efficiencies exceeding 15% in saturation mutagenesis assays of UGA OPRL1 readthrough in HEK293T cells (Figure 4 in Ref. [27]). Additionally, in the type III MoMLV assay in RRL, the wild-type viral nucleotides +8G and +9U—distinct from the type I motif—are optimal in the presence of the downstream H-type pseudoknot structure [61,63]. The positional window contributing to functional convergence may extend beyond the previously defined −3 to +9 region (Figure 1B), as the moderate-level readthrough efficiency (7.8%) of the malate dehydrogenase-1 (MDH1) gene (UGACUAGAC) is increased by simultaneous, but not individual, nucleotide replacements at the −9 and −8 upstream positions or +11 and +12 downstream positions [252].

Thus, while the importance of nucleotides immediately downstream of the stop codon is evident, it remains difficult to identify any universally crucial downstream nucleotide. Rather, multiple downstream sequence contexts, often in combination with the identity of the termination codon and adjacent upstream elements, exert measurable effects on readthrough efficiency, supporting the concept of sequence ‘contexts’. Several mechanisms have been proposed to account for readthrough stimulation by downstream elements: (i) interactions between the P-site tRNA generated after readthrough and the incoming tRNA decoding the subsequent codon [289], (ii) interactions between downstream mRNA and the ribosomal protein uS3, which occupies the mRNA entry pore and extends toward the region downstream of the A site (yeast uS3 mutational analysis [290]), and (iii) sequence-dependent interactions between mRNA and the ribosomal mRNA entry channel [288]. Consistent with the proposed mechanisms, interactions between ribosomal proteins and downstream mRNA positions have been observed within the mRNA entry channel in a recent cryo-EM study of viral F2A (PDB 9RHU [291]). Note that eukaryotic uS3 exhibits an extended structural module projecting toward the ribosomal A site, which approaches the +5–+8 mRNA positions beyond the latch of the mRNA entry channel (numbering relative to the A-site codon) [292,293] (Figure 3E).

#### 3.3.2. Downstream RNA Structures

Downstream RNA structures and local nucleotide interactions have been reported or investigated as *cis*-acting elements associated with type II and III readthrough. As these structures differ among viruses, they are described separately along with other aspects of readthrough (Section 4).

*(1)*  *Frameshifting Structures*

(i) *Ribosome–Downstream RNA Interactions*: Since no stimulatory structures for stop codon readthrough have been analyzed in complex with ribosomes, this subsection focuses on interactions between frameshifting structures and ribosomal components, which have been investigated using structural, biochemical, and single-molecule approaches [294,295,296]. Frameshifting downstream structures captured in complex with ribosomes—including the *E. coli dnaX* stem–loop (*T. thermophilus* 70S, PDB 5UQ7 [297]), the human immunodeficiency virus 1 (HIV-1) hairpin (*E. coli* 70S, PDB 6VWL [298]), and the SARS-CoV-2 three-lobe pseudoknot (rabbit 80S, PDB 7Z7O [125])—revealed direct interactions between mRNA and ribosomal components. These interactions involve proteins located at the mRNA entry pore (uS4 in bacteria; uS3 and uS5 in both bacteria and rabbit; eS30 in rabbit), as well as components on the ribosomal outer surface (uS3 in both bacteria and rabbit; eS10 together with 18S rRNA h16 in rabbit). Notably, frameshifting downstream structures can impede translation by dynamically rearranging tRNA sites while interacting with the outer surface of uS3 [297], or by forming stable interactions with the A-site finger in both the rotated and non-rotated ribosomal states [298]. The handling of such downstream RNA structures by lysine and arginine residues near the mRNA entry pore likely constitutes a front-line sensing system of elongating ribosomes, with some of its functions evidenced as helicase-like activities in the *E. coli* proteins uS3 and uS4 [299,300].

In SARS-CoV-2 structures (Figure 3E), uS3 Arg117, uS5 Arg152, and eS30 Lys125 are positioned near viral nucleotides +11, +10, and +9, respectively, relative to the A-site codon [125]. eS30 is a predominantly eukaryotic ribosomal protein that spans the mRNA entry region near uS4 and extends toward the decoding center, following the contour of h18 and contacting uS12 [119,121,125]. In mammals and other holozoans, eS30 is synthesized as an N-terminal fusion protein with FUBI, a 133-residue ubiquitin-like protein in humans. It is proteolytically cleaved during pre-40S ribosome maturation, yielding a short C-terminal eS30 polypeptide (~59 aa; residues 75–133) [301,302]. In budding yeast, RPS30 encodes a standalone eS30 protein (63 aa), obviating the need for FUBI-mediated processing [303].

(ii) *Downstream Structure Intermediate States*: Ribosome–mRNA interactions can influence the conformational properties of downstream RNA elements. SHAPE (selective 2′-hydroxyl acylation analyzed by primer extension) reactivity at a proximal base pair in the *dnaX* stem–loop RNA increases upon binding to the 70S ribosome, consistent with the cryo-EM structures (PDB 5UQ7 and 5UQ8 [297]). In the SARS-CoV-2 structure [125] (Figure 3E), the pseudoknot appears to initiate two nucleotides downstream at the +14 position. This observation is consistent with an intermediate state in which upstream base-pairing interactions are destabilized, although the timing and mechanism of partial unwinding remain unresolved.

A similar arrangement of downstream RNA elements has also been proposed for the functionally active form of the MoMLV pseudoknot, based on biochemical rather than cryo-EM data (Section 4.1.3). These observations suggest that ribosome interactions with downstream RNA elements may generally induce intermediate states that facilitate recoding events such as frameshifting or stop codon readthrough.

*(2)*  *Fidelity Control by Ribosomal Proteins along the mRNA Channel*

Several ribosomal proteins are positioned along the mRNA path in the mammalian ribosome [293,304]. Among these, mutations in uS3, eS30, and uS19 have been implicated in translational fidelity. Fidelity-related effects associated with mutations in uS4 and uS5 were discussed earlier (Section 3.1.1). uS3 and eS30 are near the mRNA entry channel and may mediate downstream mRNA-dependent effects. In contrast, eS30 and uS19 are positioned closer to the decoding center, where they likely contribute to fidelity through interactions with tRNA and the surrounding rRNA environment. Notably, eS30 spans these regions, suggesting a potential role in coupling mRNA entry with decoding fidelity.

**Figure 5:**
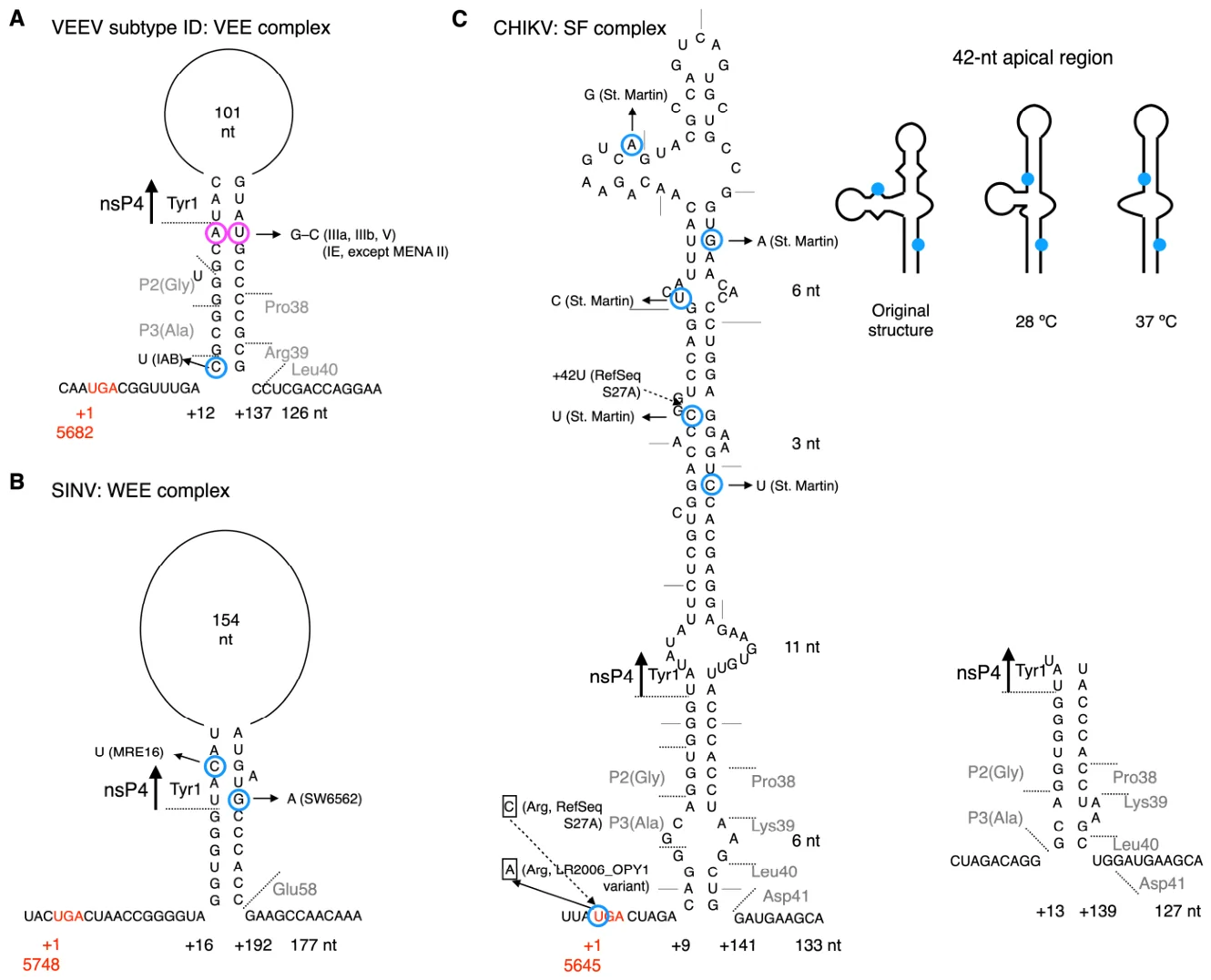
Downstream RNA structures for alphaviral UGA readthrough. (**A**) VEEV subtype ID from the VEE antigenic complex (RefSeq accession NC_001449; [337]), (**B**) SINV, the type species of the genus *Alphavirus*, from the WEE complex (RefSeq accession NC_001547; [338]), and (**C**) CHIKV strain from the 2006 Indian Ocean outbreak (SF complex; AM258994; [339]). Structural interpretations are based on [64] for (A,B) and on [67,68] for (C). Amino acid numbering is based on the nsP4 product, with the first amino acid after the cleavage site designated as amino acid 1. Where appropriate, amino acid positions corresponding to paired nucleotides are indicated in gray. The cleavage boundary generating the nsP4 N terminus is marked with a bold vertical arrow. Solid arrows indicate reported nucleotide variations among strains [64,67]; dotted arrows indicate additional variations identified in this study by comparison with the CHIKV RefSeq sequence (accession NC_004162; [340]). Variation sites are indicated by circles, with single and covariant variations shown in cyan and magenta, respectively; amino acid changes are marked with rectangles on the affected nucleotides. Except for the two nucleotide variations at +1 in (C), which alter the readthrough stop codon, all indicated variations are synonymous. In (**C**), the nucleotide-level structure originally determined by SHAPE analysis in [67] is shown on the left. The structures shown on the right are from [68], in which the basal-region conformation at 28 °C matched that shown on the left, whereas different conformations were observed in the apical region at both 28 °C and 37 °C, and an alternative conformation was observed in the basal region at 37 °C. The stop-codon-related red annotations follow those in Figure 4A. For Figure 5 and Figure 6, nucleotide positions are numbered from the U of the UGA stop codon as nucleotide +1, and bars indicate 10-nt intervals. The GenBank accession numbers for the MENA II, MRE16, SW6562, and St. Martin strains are AF075252 [341], AF492770 [342], AF429428 [343], and MT228631 [344], respectively. The nonsense-to-sense substitution is based on the pCHIKV-LR infectious clone (EU224268 [345]). CHIKV, chikungunya virus; SF, Semliki Forest; SINV, Sindbis virus; VEEV, Venezuelan equine encephalitis virus; WEEV, western equine encephalitis virus.

As described, eukaryotic uS3, which extends inward toward the ribosomal A site, affects translational fidelity when the two basic residues located at the mRNA entry pore are mutated [290]. Similar effects on decoding have been reported for uS3 in bacteria [299]. Yeast genetic studies revealed that the N-terminal region of eS30 (up to 10 amino acids) contributes to near-cognate readthrough (Figure 5C and extended data Figure 5 in Ref. [305]), including His5 (His77 in humans), positioned near the decoding center in mammalian 80S structures [119,121,133]. Additionally, near the ribosomal head side, uS13 in bacterial ribosomes directs its C-terminus toward the P-site tRNA [306]. In contrast, in eukaryotic ribosomes, uS19 (145 aa in humans) occupies a similar position but directs its C-terminus toward the decoding center [119,121,307,308,309]. Several fidelity-related single-point mutations in residues 134–138 (GATHS), a region that interacts with the P-site tRNA anticodon stem (PDB 6Y0G [307]), have been reported in patients with chronic lymphocytic leukemia [310]. Notably, premature death syndrome in *Podospora anserina*, a filamentous fungus, has been reported to be caused by mutations corresponding to uS19 Gly139Asp or Gly139Cys (equivalent to substitutions at Gly132 in humans), suggesting that this region contributes to translational accuracy [311], with additional mutations affecting mitochondrial genome stability and cellular stress responses contributing to the observed phenotype.

eS30 His77 forms a close contact with the A-site tRNA near the 36–37 tRNA backbone region in the GTPase activation (GA) state [119,133] (Figure 3F), suggesting a role in stabilizing cognate A–minor interactions [121]. This interaction is stabilized by the contact between eS30 Lys75 and A1825, although these contacts are not resolved when A-site tRNA is absent from density or in the early codon recognition (CR) state [119,133]. These contacts are not consistently resolved across accommodated (AC) structures, likely reflecting local conformational variability. Notably, a recent cryo-EM analysis of a rabbit 80S translation complex observed a proximity between the N-terminal region of eS30 and the tRNA anticodon stem backbone (PDB 9Q2P) in a state proposed to be associated with kinetic proofreading [312]. This structural feature may indicate a potential contribution of the eS30 N-terminus to the geometric inspection of tRNA during elongation, although the precise mechanistic implications remain to be fully established.

The structure of the C-terminal region of uS19 is not well-resolved in the CR or GA states of elongating or terminating ribosomes [119,121]. Nevertheless, interactions of Lys145 and Pro143 with the backbones of 18S rRNA h44 and the A-site tRNA anticodon loop have been observed in the AC state [119] (Figure 3G), suggesting potential stabilizing effects during kinetic proofreading. In the subsequent PRE state before translocation, Lys145 is repositioned toward the decoding center, where it interacts with multiple nearby elements, including the 18S rRNA backbone near A1825 and G1826, and the 28S rRNA nucleotide Am3760 [307], which itself approaches the tRNA backbone near position 37 in the GA state [119]. With Pro143 contacting the A-site tRNA backbone at position 38, these geometries support a role in stabilizing tRNA positioning, which may in turn contribute to decoding and maintenance of the reading frame.

Eukaryote-specific structural elements are observed in ribosomes decoding cognate codons. These stabilizing interactions likely contribute to the slower kinetics and higher fidelity of eukaryotic canonical elongation compared with bacteria. Several fidelity-related mutations in these proteins [310], located remote from the decoding center, suggest that fidelity is controlled by factors outside the A site, including P-site codons and downstream elements, possibly mediated by these proteins (including eS30). The roles of these proteins in near-cognate elongation remain to be elucidated.

Together, downstream RNA structures engage the ribosome at multiple contact points, including the mRNA entry channel and adjacent ribosomal regions. These interactions, often mediated by proteins such as uS3 and eS30, influence decoding by modulating mRNA trajectory and dynamics. Importantly, the RNA elements are dynamic, undergoing progressive unwinding and rearrangement during translation, potentially affecting ribosome pausing, tRNA positioning, and the balance between termination and near-cognate elongation.

## 4. A Specific Review of Readthrough Sites in the Four Viral Families

In this section, nucleotide numbering generally follows the original studies. Positions relative to the termination codon are indicated with the prefix ‘+’.

### 4.1. Retroviral UAG Stop Codon Readthrough

In the family *Retroviridae*, *pol* gene expression strategies differ among genera. In the subfamily *Orthoretrovirinae*, the *pro* and *pol* genes are translated from the upstream *gag–pro–pol* transcript, but their expression is restricted by an in-frame stop codon located within the *gag–pro–pol* coding region. In gammaretroviruses and epsilonretroviruses, translation of the downstream *pro* and *pol* genes relies on stop codon readthrough, whereas in alpharetroviruses, betaretroviruses, deltaretroviruses, and lentiviruses, it is achieved by programmed frameshifting. Alpharetroviruses generally retain the canonical retroviral gene organization. Some gammaretroviruses additionally express glycosylated Gag (glyco-Gag) from an upstream initiation codon, as exemplified by the CUG-initiated glyco-Gag of MoMLV [313]. In contrast, epsilonretroviruses contain distinct additional ORFs outside the canonical *gag–pol* and *env* genes. Glyco-Gag provides an accessory function that enhances viral infectivity by antagonizing host retroviral restriction factors, particularly serine incorporator 5 (SERINC5), in a manner analogous to HIV-1 Nef [314,315], and has also been implicated in protection against APOBEC3-mediated restriction [316,317]. Viral replication in gammaretroviruses and epsilonretroviruses can depend on efficient stop codon readthrough at the *gag*–*pro* junction.

The type species of the genera *Gammaretrovirus* and *Epsilonretrovirus* are MoMLV and walleye dermal sarcoma virus (WDSV), respectively, the latter being isolated from walleyes in the USA.

The junctional stop codon between the *gag* and *pro* genes is downstream of the zinc-finger domain (the Gag knuckle) within the nucleocapsid (NC) region of Gag and upstream of the catalytic DTGA motif of the aspartic protease Pro. The exact location of the stop codon differs between gammaretroviruses and epsilonretroviruses. In MoMLV (a gammaretrovirus), it is located at the fifth amino acid residue of the protease, whereas in WDSV (an epsilonretrovirus), it is located at the penultimate residue of NC [318].

#### 4.1.1. Gammaretrovirus

**Figure 4:**
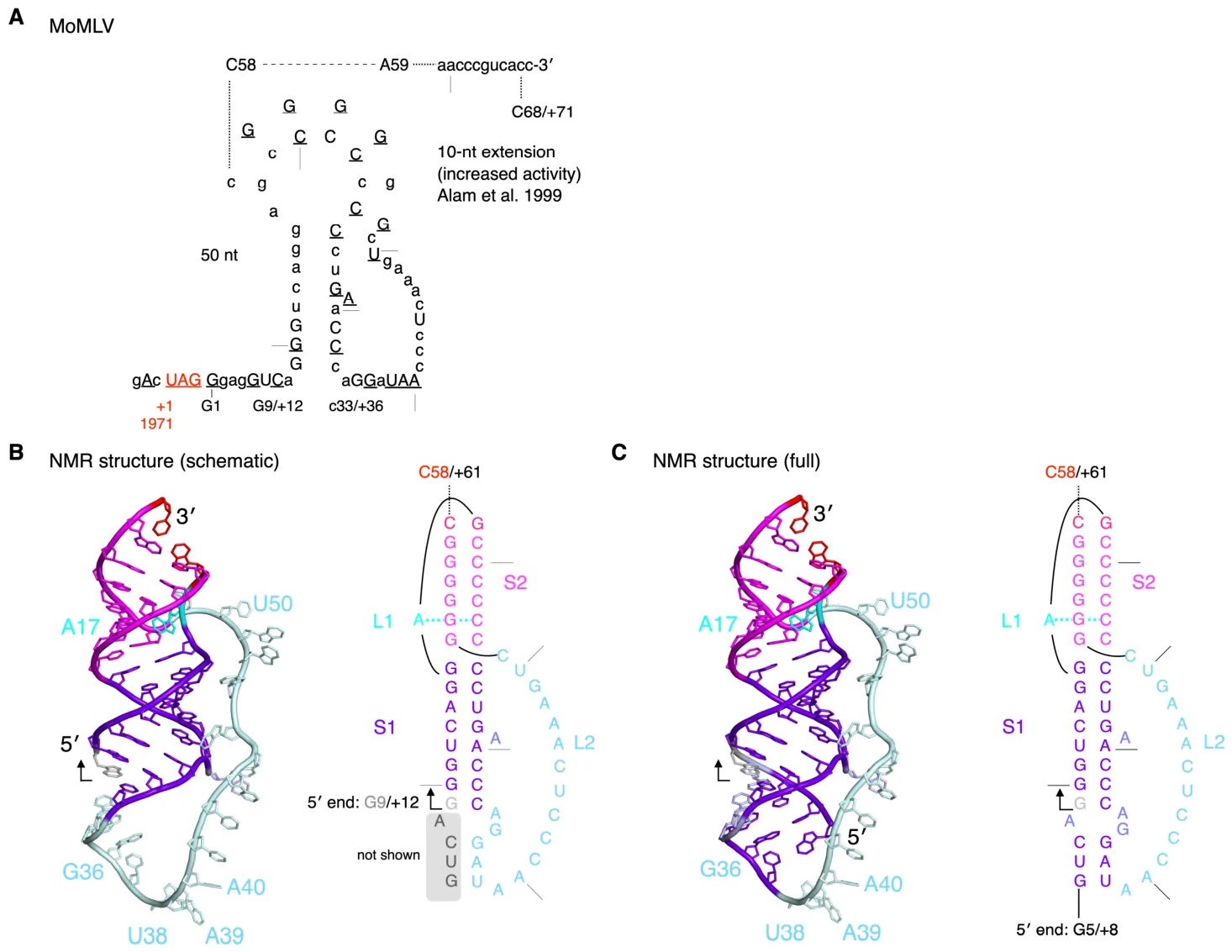
Gammaretroviral downstream stimulatory RNA structures. Tertiary structure model (**A**) and solution NMR structures (**B**,**C**) of the gammaretroviral MoMLV readthrough pseudoknot. (**A**) The biochemically supported active-form pseudoknot model, including a spacer and an H-type pseudoknot, is aligned based on functional assays [61,63,94], with conserved nucleotides shown in uppercase as defined in [63]. Nucleotides are underlined if conserved across the 19 representative gammaretroviral sequences analyzed in Table 2 and shown in lowercase otherwise. In the main text, conserved nucleotides are simplified and shown in bold for clarity. Elements shown in red indicate the termination codon, its reference position (+1 relative to the termination codon), and the corresponding viral nucleotide number. Nucleotide positions are indicated every 10 nt with bars for clarity, starting from +4 relative to the termination codon at the readthrough site, corresponding to the scale used for MoMLV. Dotted lines indicate the nucleotide positions marking the 3′ end of the pseudoknot (C58), the following nucleotide (A59), and the downstream position (C68/+71) reported to be associated with increased readthrough activity by Alam et al. (1999) [94]. Dashed lines indicate continuity of the RNA sequence between C58 and A59, which are separated in the schematic representation. (**B**,**C**) NMR solution structures (PDB 2LC8 [97]). The stem (S1, S2) and loop (L1, L2) regions are color-coded as shown in the conventional pseudoknot topology diagram. In panel B, four nucleotides in the spacer, GUCA (G5–A8, shaded in the diagram), which are base-paired with 36GAU in L2 in the solution structure (C), are omitted, as these base pairs are dispensable for activity [63]. The 5′- and 3′-terminal nucleotides of the biochemically supported active-form pseudoknot, corresponding to G9 and C58 shown in panel A, are colored gray and red, respectively, together with G18, which is paired with the 3′-terminal nucleotide C58. Functionally relevant bases in the L1 and L2 regions are labeled, and the A17–23C–G53 base triple is indicated by a cyan dot in the diagram. MoMLV, Moloney mouse leukemia virus.

The H-type gammaretroviral pseudoknot, comprising 50 nucleotides in MoMLV, is preceded by a functionally important 8-nucleotide purine-rich spacer enriched in guanosine residues at its 5′ end, with conserved nucleotides at positions G1 and G5 [61,62,63] (Figure 4A). Different RNA structures were predicted in the *gag–pro* junction region, with starting points ranging from approximately −18 to +12 nucleotides relative to the termination codon, allowing partial overlap among proposed elements [61,62,76,83,287,319]. The involvement of the pseudoknot, predicted to initiate at nucleotide +12 (G9), was verified by 3′-side deletion and compensatory mutations in stem 1 (S1) and stem 2 (S2). The starting point of S1 has been identified as the **G**9–C33 pair, because the functional importance of conserved single-stranded bases in the spacer (**G**1, G2, **G**5, **U**6, **C**7, and A8; the conserved nucleotides are shown in bold) and loop 2 (**G**35, **G**36, **U**38, **A**39, and **A**40 in L2) regions has been demonstrated by mutagenesis [61,63,94] (in Section 4.1.1 only, double-underlined bases indicate nucleotides conserved among all nineteen species in Table 2).

With respect to the 3′ end, subsequent cell-free analyses revealed stimulatory effects of an additional 11 nucleotides, including C58 at the 5′-most position of the segment, which forms a base pair with G18 at the 3′ end of S2 [94,97]. Related 3′ sequences were included in later functional assays [67,99] (Table 3).

The presence of seven G–C Watson–Crick base pairs in the tertiary structure of MoMLV distinguishes it from other reported pseudoknot structures, including viral frameshifting pseudoknots such as those in infectious bronchitis virus (IBV) [320], yeast L-A virus [321], and others [322], as well as gammaretroviral readthrough pseudoknots from some species (Figure 4A).

As a polypeptide, the RNA element corresponding to the spacer and pseudoknot encodes the N-terminal fold of the protease. The crystal structure of this protease fold from the laboratory-derived xenotropic murine leukemia virus-related virus (XMRV; PDB 3NR6) has been determined [323,324]. This protease is highly homologous to that of MoMLV and consists of 126 amino acids, with two amino acid substitutions (Asp3Gly and Met122Val). Although the six consecutive guanylates on the 3′ side of S2 encode a diglycine motif, structural analysis indicates that a turn-like conformation can still be adopted in the absence of the diglycine motif. Instead, the triproline motif encoded by the opposing cytidylate-rich strand influences whether the N-terminal fold is positioned above or below the main body of the protease (e.g., Figure S4 in Ref. [323]). These seven consecutive G–C pairs and the triproline motif, characteristic of mouse leukemic gammaretroviruses, are not strictly required for readthrough, as substituting a single G–C pair with A–U in GaLV retains substantial activity [63]. Early MoMLV mutational analyses also indicated that alterations affecting the S2 stem did not always abolish readthrough activity [61]. The functional significance of this N-terminal fold for protease orientation and substrate processing has not yet been determined, and further studies are warranted to clarify its role.

#### 4.1.2. Epsilonretrovirus and Unclassified Retroviruses

Stop codon readthrough in epsilonretroviruses has not been experimentally verified. Information on the stop codon context is currently limited to WDSV, and the nature of the *cis*-acting elements remains unclear. In WDSV, the readthrough site is located at the C-terminus of the NC protein. Although the identity of the incorporated amino acid(s) has not been determined, glutamine has been predicted because the readthrough codon corresponds to the P2 position of the Gag–Pro cleavage site, a position recognized as glutamine by WDSV Pro [318].

In one unclassified retrovirus and two endogenous retroviruses that are distantly related to epsilonretroviruses (Table 2), the termination codon is UGA instead of UAG. Hexanucleotides starting from the termination codon include UGAAGA in exogenous Atlantic salmon swim bladder sarcoma virus (SSSV) [325] and UGACGA and UGAAGU in two endogenous retroviruses—one from zebrafish [326] and the other from a frog [327], respectively. In contrast, the unclassified retrovirus, snakehead retrovirus (SnRV) [328], has the hexanucleotide UAGGGC, differing by a single nucleotide at the +6 position from the type III gammaretroviral UAGGGA motif of MoMLV. These data, which include at least two exogenous viruses [325,328], indicate that retroviral readthrough is not restricted to a specific sequence context and can occur across diverse aquatic hosts.

#### 4.1.3. H-Type Pseudoknot in Gammaretrovirus

Several structural features underlying readthrough have been investigated using a proposed active conformation, building on earlier comparative analyses [61] and derived from conserved nucleotides across five representative gammaretroviruses (BaEV [baboon endogenous virus], FEV, GaLV, MoMLV, and REV) [63] that has been refined by structural mapping [94] and NMR (PDB 2LC8 [97]), as described below (i–iv).

(i) *Structural Stability*: Mutations disrupting base pairing in S1 or S2 have been reported to reduce readthrough efficiency [61,62,94,97,99]. In contrast, a synonymous mutation that converts the 12U–30A pair to 12C–30G on S1 [99] increases readthrough efficiency in cultured cells by approximately twofold, suggesting that the wild-type sequence conformation may still have room for further enhancement.

Conversely, mutations that convert the 15G–26C pair to 15A–26U on S1 [97] reduce efficiency by twofold, indicating that structural integrity, in terms of stability and geometry, contributes to readthrough activity.

(ii) *Unpaired Nucleotides*: Probing analyses of the MoMLV H-type pseudoknot revealed unpaired nucleotides at two positions: A17 in L1 and A34–C51 in the region referred to here as L2 (which corresponds to the canonical L3, since the original L2 contains no unpaired nucleotides). Consistent with this, solution structures reveal that A17 forms a base triple, whereas no triplex formation occurs between S1 and L2. The roles of the unpaired bases in L1 and L2 (corresponding to L3 in pseudoknots in general), as well as the single-nucleotide bulge **A**29 in S1, have been investigated through extensive mutational analyses. Three-nucleotide deletions within the L2 region are deleterious for readthrough efficiency [61,62]. Conserved nucleotides are generally required for the readthrough, and single-nucleotide substitutions at these positions reduce readthrough efficiency. Given the structural flexibility of MoMLV L2 in solution, it is conceivable that part of L2 could contact the ribosome, contributing to pausing at the recoding site. Mutations at certain positions, including **A**29C, A37C, **U**38A, and combinations thereof, increase readthrough efficiency. These observations suggest that the wild-type sequence is not fully optimized for readthrough or structural stability. Importantly, if spacer–L2 base pairing (see below) were an essential component of the active conformation, the **U**38A mutation would be expected to reduce readthrough efficiency; however, no such reduction in readthrough efficiency is observed [63,94,97,99].

(iii) *A17 base triple*: In MoMLV, A17 in L1 forms a base triple with C23–G53 from the major groove side, with G53 serving as the central base. The distance between A17 C2 and G53 O6 is 3.0 Å, consistent with protonation-dependent stabilization of the triple. In contrast to the frameshifting pseudoknot of mouse mammary tumor virus (MMTV; PDB 1RNK [329]), in which the S2 3′-terminal GU pair can deviate naturally from coaxial stacking, the all–G–C composition of MoMLV S2 precludes such structural relaxation. Consequently, the single nucleotide A17 must adopt a strained conformation spanning seven base pairs in S2. The relationship between the protonation of A17—modulated by pH—and readthrough efficiency has been investigated using NMR, directly assessing pH effects [97]. Beyond this, whether protonation of other nucleotide bases in the pseudoknot—including those in the base triple, as proposed for A17—affects readthrough efficiency remains to be elucidated.

(iv) *Spacer–L2 interactions*: Solution NMR structures (PDB 2LC8 [97]) revealed additional base pairs at the 5′ end of the pseudoknot. Base pairs between 5**GUC** on the spacer and 36**G**A**U** on L2 extend stem S1 by three nucleotides toward the stop codon, causing 34 A**G** to form a bulge on the descending strand (Figure 4B). Computationally predicted pairings include the 6**UC**–35**GG** pairing, proposed as a working model for viral replication [287], and the 34A**GG**–42CC**U** pairing within L2, proposed as part of a three-stemmed pseudoknot model [330]. The latter may be consistent with the weak bands observed in the reported enzymatic probing pattern [94], suggesting structural heterogeneity and complexity in the region connecting the spacer and S1. With respect to spacer–L2 interactions, single-point mutations predicted to stabilize the GU wobble pair reduce readthrough efficiency (e.g., **G**5A [61,63] and **U**38C [63,97]), whereas A37C and **U**38A increase efficiency, as described. These observations argue against a direct functional role for spacer–L2 base pairing in promoting readthrough (shaded in Figure 4B). Instead, these mutations suggest that the thermodynamically less stable pseudoknot conformation (Figure 4A) may represent the active form, potentially stabilized or selectively engaged during ribosome transit. The extended stem S1, resulting from the additional 5′ base pairs, may influence ribosome progression, analogous to the shift in the pseudoknot start site observed in SARS-CoV-2 [125], thereby contributing to its roadblock function. Alternatively, and not mutually exclusive with the proposed active form, RNAs formed by spacer–L2 interactions may remain base-paired, with the extension of S1 extending stacking interactions, thereby contributing to the structural integrity proposed to support readthrough activity [97]. Elucidating the interactions between downstream structures and translating ribosomes, particularly through structural studies, would clarify how these structural ensembles contribute to the formation of an active conformation that can trap ribosomes.

### 4.2. Alphaviral UGA Stop Codon Readthrough

#### 4.2.1. Phylogeny Within the Genus Alphavirus

The genus *Alphavirus* is the sole genus in the family *Togaviridae*. The genus comprises more than 30 species, including insect-specific and fish-specific species. Human and vertebrate pathogens are classified into two groups based on geographical isolation, which is also correlated with clinical symptoms: New World alphaviruses, identified in North and South America and associated primarily with encephalitis, and Old World alphaviruses, identified in the Old World, including Australia and the Oceanian islands, and closely related to arthritis and rash.

The genus name *Alphavirus* derives from the term “group A arbovirus”. Most alphaviruses associated with vertebrate disease are transmitted by arthropods, typically through mosquito bites. The replication cycle has been best characterized in the New World alphavirus EEEV, isolated from North America and maintained between birds and bird-preferring *Culex* mosquitoes. Humans and horses are migratory hosts that suffer severe symptoms, with transmission by mammal-preferring *Aedes* mosquitoes.

Downstream RNA structures have been studied mainly in three viruses: SINV in the WEE complex, VEEV in the VEE complex, and CHIKV in the SF complex (Figure 5).

#### 4.2.2. Alphaviral Readthrough Sites

The alphaviral UGA readthrough termination codon is located in the junction region between nsP3 and nsP4. nsP3 is a multifunctional phosphoprotein, whereas nsP4 functions as the viral replicase [331,332,333,334]. This codon lies downstream of the nsP3 hypervariable domain (HVD) [335,336] and seven codons upstream of the conserved cleavage site between nsP3 and nsP4: P2(Gly)–P1(Ala/Gly) | P1′(Tyr)–P2′(Ile)–P3′(Phe) (the cleavage site is indicated by |, see Figure 5 for partial mapping of P3, P2, and P1′ residues). While the nsP3 HVD is highly variable in sequence and length, the six-amino-acid readthrough extension that forms the N-terminal portion of the cleavage site is conserved within subgroups of serological complexes.

As described in Section 2.5, alphaviruses use two different type II hexanucleotides. UGACGG occurs in VEE, EEE, and a subset of WEE-related complexes (WEEV-like group), and is absent from Old World isolates. In contrast, UGACUA occurs in SF and a subset of WEE-related complexes (SINV-like group), and is distributed across both New and Old World isolates. Synonymous variations among New World viruses include UGACGA in Rio Negro virus (RNV, a subtype of VEEV, VEEV-VI), UGACGC in Mosso das Pedras virus (MDPV/VEEV-IF) and Fort Morgan virus (FMV), UGACUG in Trocara virus (TROV), and UGACUU in Caainguá virus (CAAV). Thus, the alphaviral hexanucleotide can be represented as UGACGV or UGACUD, where ‘V’ and ‘D’ denote any nucleotide except U and C, respectively. These observations suggest that readthrough may occur in several different sequence contexts within alphaviruses.

#### 4.2.3. Alphaviruses with Nonsense-to-Sense Codon Changes

In contrast to retroviruses, in which the presence of the *gag–pol* junctional termination codon defines the family, some alphaviruses encode a sense codon at the corresponding readthrough site [85,346]. Alphaviruses are instead classified based on sequence homology across full-length genomes rather than the presence of the readthrough codon.

Among the distantly related alphaviruses with host ranges distinct from mammal- or bird-associated alphaviruses, insect-specific alphaviruses (Table 2), isolated from mosquitoes and unable to replicate in known vertebrate cells, such as Eilat virus (EILV [347]) and Taï Forest alphavirus (TFAV [348]), retain the canonical UGACUA motif and likely utilize stop codon readthrough for nsP4 expression [349,350,351]. In contrast, salmonid alphaviruses (SAVs) and related alphaviruses isolated from fish, such as salmon pancreatic disease virus (SPDV [352]) and sleeping disease virus (SDV [353]), encode alternative sequences at the corresponding positions (e.g., CAACGG or CAACGU) [354,355,356,357] and are thought not to rely on readthrough, with Wenling fish alphavirus as an exception (Table 2) [358]. These observations indicate that alphaviral strategies for polymerase expression are more flexible than those of gammaretroviruses and may be shaped by host range, viral lineage, or both.

Within the SF complex and SINV, as well as two additional strains that utilize the UGACUA readthrough motif, variants encoding sense codons have been isolated, most commonly CGA, with AGA, UGG, UGC, and UGU also observed (Table 2). In addition, the GenBank reference sequences for SFV (NC_003215 [359]) and CHIKV (NC_004162 [340]) encode CGA (Arg).

Although the precise biological significance of nonsense-to-sense codon changes in alphaviruses remains unclear [333], several factors may contribute to their emergence:

(i) *Quasispecies at the readthrough site*: Investigations of the origin of o’nyong-nyong virus (ONNV) variants from the 1996–1997 epidemic revealed quasispecies at the readthrough site, including UGA, CGA (Arg), and possibly other sense codons, during passage in Vero cells [360]. A viral population in the host has been suggested to exist in equilibrium between opal and sense codons [73]. Additionally, early mosquito samples typically consisted of pooled mosquitoes.

(ii) *Selection for Sense Codons during Passaging*: As considered in previous studies [64,346], viral passaging prior to cloning may impose selective pressure at the readthrough site. Evidence for this has been obtained during ONNV passaging in Vero cells [360] and during EEEV passaging in mosquito C6/36 cells [361]. Conversely, during replication in natural mosquito hosts, ONNV carrying the opal codon disseminates more efficiently than the virus carrying the CGA sense codon [73].

(iii) *Properties of the nsP4 Replicase Complexes*: Modulation of nsP2 protease cleavage sites promotes early RNA synthesis of SFV [362] and also alleviates reduced replicon activity caused by RRV nonsense-to-sense codon changes in vertebrate cells [85], although these effects are temperature-sensitive. These observations suggest that alphavirus RNA synthesis relies on a finely tuned balance between nsP4 replicase production and viral polypeptide processing. This system may be sensitive to perturbations but also confer plasticity that allows replication in alternate hosts. Switching between readthrough and sense codons at the nsP3 terminus may further expand this adaptability, with the opal codon providing an effective regulatory solution. The stability of nsP4 replicase, which has been characterized as unstable in SFV [363,364], may contribute to the emergence of viruses with nonsense-to-sense codon changes.

(iv) *Viral Adaptation to New Hosts*: Viruses with nonsense-to-sense codon changes have been isolated from patient-derived samples of RRV [365], ONNV [360,366], and CHIKV [340,367,368,369,370]. Additional RRV and CHIKV sequences carrying nonsense-to-sense codon changes have been reported but remain uncharacterized. While the mechanistic basis remains unclear, such variants may reflect viral adaptation to distinct vertebrate host environments, a notion supported by in vivo studies: an opal-to-Arg CHIKV clone showed an attenuated disease phenotype in a mouse model of arthritis [369,371], suggesting that nonsense-to-sense changes can modulate viral fitness during host infection.

An infectious clone derived from the La Réunion isolate (EU224268, AGACUA [345]) differs from the reported sequence of LR2006_OPY1 (DQ443544, UGACUA [372]), consistent with mutations occurring during post-isolation handling.

These observations indicate that the emergence of viruses with nonsense-to-sense codon changes may reflect both naturally occurring sequence variation within infected hosts and subtle changes introduced during laboratory propagation, as well as the interplay between replicase dynamics, translational regulation at the nsP4 junction, and viral adaptation to diverse host environments.

#### 4.2.4. Alphaviral Downstream Extended Stem–Loop Structures

The extended stem–loop structures of VEEV and SINV, which are 126 and 177 nt long, respectively, rely on base pairing between two phylogenetically conserved nucleotide sequences separated by 101 and 154 nt, respectively. Sequences proximal and distal to the UGA readthrough site encode amino acids in the nsP3–nsP4 cleavage site and at positions 37–40 and 54–57 in the N-terminal domain (NTD) of the nsP4 replicase. Within the same antigenic complex, or within individual subgroups thereof, these nucleotide sequences are typically conserved in sequence [64]. Even synonymous mutations are rare and, when present, are accommodated by alternative base-pairing interactions or occur as covariant mutations that preserve the overall RNA structure (Figure 5).

The VEEV and SINV structures increase readthrough efficiency by 9.5- and 3.2-fold, respectively, in HEK293 cells, with the effect of the former confirmed by a destruction–restoration assay [64]. Enhancement in SINV may depend on the basal readthrough level and cell type, as the structure increases efficiency by 1.5- and 2.4-fold in Vero and C6/36 cells, respectively, where baseline readthrough reaches nearly 10% [85].

Base pairing in VEEV is largely conserved in EEEV and WEEV [64], with GC-rich nucleotides encoding P3(Ala)–P2(Gly) paired with CG-rich sequences encoding nsP4 Pro38–Arg39 (CGC) (Figure 5A). In CHIKV, substitution of Arg39 (CGC) with Lys39 (AAG) disrupts this pairing pattern, resulting in alternative pairing conformations involving nucleotides encoding Leu40 (CUG). The structure of the CHIKV 2006 Indian Ocean outbreak strain, reported by two groups based on SHAPE analysis, is a 133-nt extended stem–loop with two forks in the apical loop (Figure 5C, +9C–+141G) [67,68]. Notably, the 5′ fork can form alternative base pairs. An alternative conformation, inferred from SHAPE analysis, has also been reported for the basal region, forming a shorter 127-nt structure (+13G–+139C) [68]. CHIKV readthrough efficiency depends on its termination codon [67,68], consistent with observations in other UGA readthrough systems (SINV, CTFV, and PSIV; Section 3.3.1, (2) Stop Codon Identity). However, the effects of the downstream structure on readthrough have neither been investigated [67] nor confirmed in viral replication-based assays [68], leaving its functional significance unresolved. How these structurally diverse alphaviral downstream RNA elements contribute to readthrough efficiency, potentially involving conformational dynamics as observed in the CHIKV apical region, remains to be clarified.

### 4.3. Coltiviral UGA Stop Codon Readthrough

Coltivirus is one of the nine genera in the subfamily *Spinareoviridae*, which, along with *Sedoreoviridae*, forms the family *Reoviridae*, a group of non-enveloped, class III double-stranded RNA viruses. The host range of reoviruses is wide; in addition to a broad range of animals, some genera also replicate in fungi, protozoa, and plants. Their genome is segmented into ten to twelve parts, and translation uses 5′-capped, 3′-non-polyadenylated mRNA transcripts.

#### 4.3.1. Segment 9 Readthrough Sites

Coltiviruses are arboviruses transmitted between ticks and large vertebrate hosts such as hares. The twelve-segmented genomes of six coltivirus species have been fully sequenced (Table 2). Some are known human pathogens [373,374,375]; for example, CTFV and Eyach virus (EYAV) cause tick-borne fever and neurological diseases in the western U.S. and Europe, respectively. Although California hare coltivirus (CHCV) is listed in GenBank as a CTFV strain, phylogenetic analysis places it closer to EYAV [375].

In segment 9 of the twelve-segmented coltivirus genome, the type II hexanucleotide UGACCG mediates readthrough, producing the structural protein VP9 (338 aa in CTFV) [376] and its C-terminal extension, VP9′ (602 aa), for which no homologues have been identified. Nevertheless, both virological [377,378,379,380] and metagenomic [381,382] analyses have increasingly identified RNA sequences corresponding to segment 9 and other segments, indicating greater genomic diversity of coltiviruses than previously appreciated.

#### 4.3.2. Coltiviral Extended Stem–Loop

**Figure 6:**
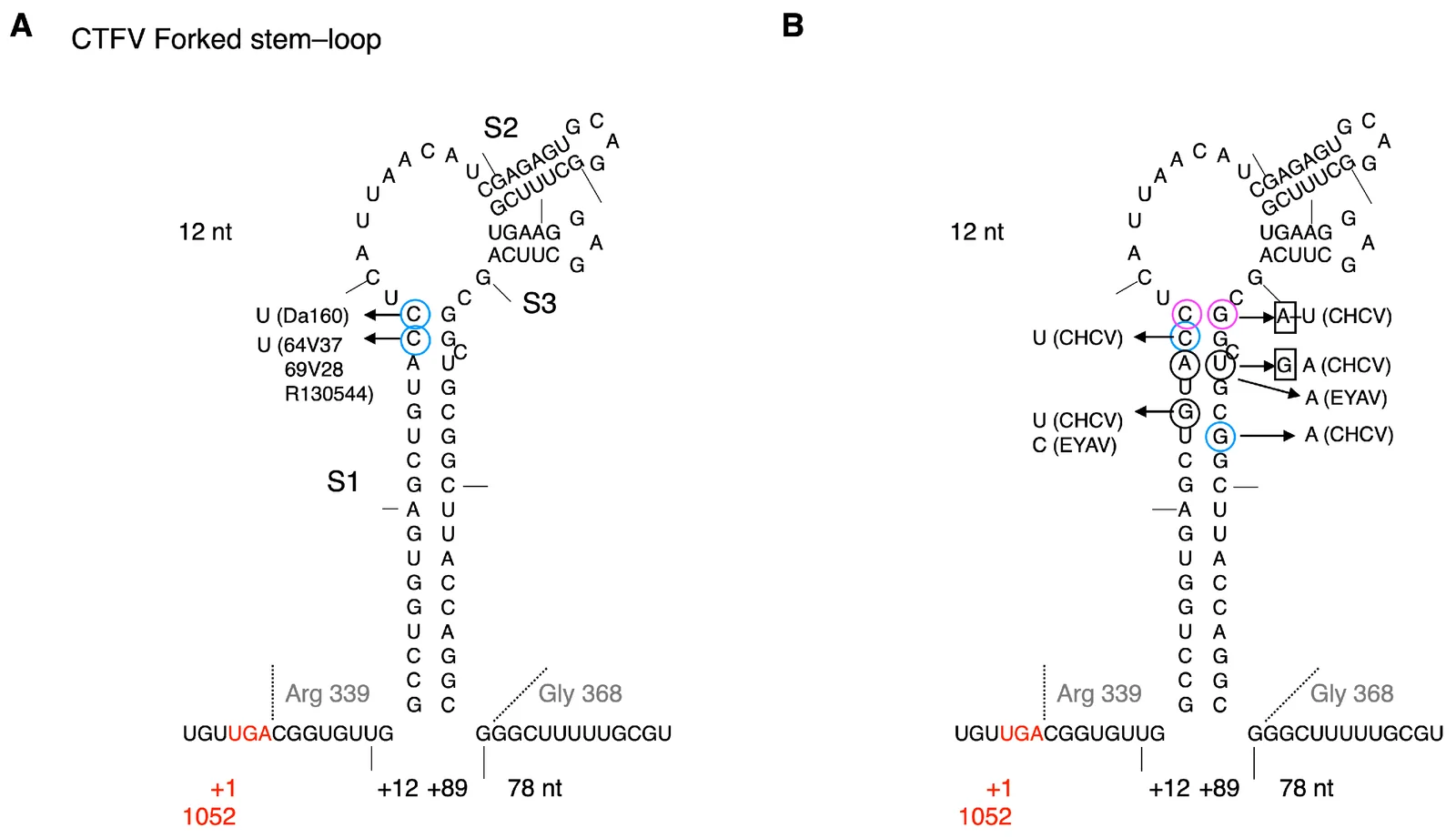
Downstream RNA structures for coltiviral UGA readthrough. Primary and secondary RNA structures of CTFV segment 9 RNA are shown schematically with variations within CTFV (**A**) and between distantly related EYAV and CHCV (**B**), with structural interpretations based on [66]. The stop-codon-related annotations, arrows, circles, and nucleotide numbering are as in Figure 5. Variations in (**B**) that disrupt base pairs are indicated by black circles, whereas additional variations outside S1 are omitted for clarity. Amino acid numbering follows the ORF1 numbering of CTFV segment 9. CTFV strain accession numbers include NC_004180 [385] (VR–90, reference strain), OQ833554 (64V37), AF007182 [386] (69V28), KY454216 [383] (Da160), and MZ484454 [375] (R130544, also known as Salmon River virus). Those for EYAV and CHCV are NC_003704 [373] and MW396988 [375], respectively. Variations shown in this figure are synonymous except for A+26G (Tyr346Cys) and C+28A (Leu347Met) in CHCV. CHCV, California hare coltivirus; CTFV, Colorado tick fever virus; EYAV, Eyach virus.

Readthrough in CTFV requires a 78-nt downstream RNA structure starting at +12G after the termination codon [66] (Figure 6A). The structure, verified by enzymatic footprinting [66], appears highly conserved among CTFV isolates and the related Salmon River virus based on extensive sequence conservation [375,383], and is essential for efficient readthrough. It contains a basal stem (S1), a large single-stranded loop, and two distal stem–loop structures. In CTFV, the S1 stem (17 bp) represents the longest viral stimulatory stem identified thus far in animal viruses. Destabilizing mutations in the distal region of S1 had little effect on readthrough efficiency in RRL. Together with lead(II) probing data and naturally occurring synonymous substitutions at positions +27 and +28 (Figure 6A), these observations suggest that the distal portion of S1 may be structurally flexible and less important for readthrough stimulation than the proximal stem region.

In distantly related EYAV and CHCV, several additional mutations disrupt base pairs in the distal portion of the basal stem (Figure 6B), while a 12-base-pair core is retained. This conservation of the proximal stem region may indicate that it plays a more important role in readthrough stimulation than the distal portion. Consistent with this interpretation, conversion of the central G+19:U+82 wobble pair to a G+19–C+82 base pair increased readthrough efficiency 2.2-fold in HEK293T cells and 1.6-fold in RRL.

In RRL assays, UGACGG performs better than UGACUA in the presence of the downstream structure [66], with UGACAG being the most efficient hexanucleotide tested. In a related RRL-based assay system with edeine, the frameshifting IBV pseudoknot has been shown to pause ribosomes, an effect that has been proposed as a key function of the pseudoknot in frameshifting [320,384]. Compared with the IBV pseudoknot, which generates paused products during its characteristic pausing window [66], CTFV mRNA with a CGA sense codon at the readthrough site produces fewer translational products, including paused products, suggesting that the CTFV extended structure acts through a distinct mechanism, possibly more closely linked to the preceding stop codon.

### 4.4. Dicistroviral UGA Stop Codon Readthrough

The family *Dicistroviridae*, one of eight families in the order *Picornavirales*, comprises small, non-enveloped, class IV (+)-strand RNA viruses. Their genomes resemble those of the family *Picornaviridae*, with a VPg-linked 5′ end and a polyadenylated 3′ end [387,388]. However, the reading frames are divided into two, separated by an IGR, with capsid-coding ORF2 located downstream (Figure 2). The VP2 initiation codon at the 5′ end of ORF2 is non-AUG: CAA (Gln) in PSIV, GGC (Gly) in Israeli acute bee paralysis virus (IAPV), and GCN (Ala) in most other species [387,389]. Capsid translation is driven by an internal ribosomal entry site in the IGR (IGR–IRES) between two ORFs, which functions without initiation factors or initiator tRNAs [390,391,392]. Subsequent studies have demonstrated that this non-AUG initiation mechanism involves an initial eEF2-mediated translocation step [393,394,395,396], followed by the recruitment of elongator tRNA [397,398,399,400]. Based on the structural properties of IGR–IRES and virion, the family is currently divided into three genera: *Aparavirus*, *Cripavirus*, and *Triatovirus*.

#### 4.4.1. Dicistroviral Readthrough Sites

Dicistroviral ORF1 stop codons and their locations vary among species [69], making readthrough events in this genus generally sporadic. PSIV [401], however, represents a clear example of stop codon readthrough, which has been experimentally confirmed in mammalian systems and Sf9 cell-free extracts [69,398]. While a stretch of hydrophobic residues is encoded within an 186-nt IGR, its role in the viral life cycle has not yet been experimentally characterized. Analysis of viral ORF architectures in related viruses reveals that some species, such as TrV, possess a frame that avoids in-frame stop codons over 180 nt. Several putative dicistrovirus-like viruses identified from aquatic or aquatic-associated samples exhibit even shorter intergenic regions of 120–123 nt that lack in-frame stop codons and may extend into ORF2 by readthrough.

TrV, the type species of *Triatovirus* isolated from triatomine bugs of the genus *Triatoma* [402], the genome of which has been sequenced [403], contains an extended reading frame within the IGR at the end of the 3D polymerase coding region. The TrV IGR-associated reading frame follows the UGAUCU nucleotide sequence at the end of the 3D polymerase coding region and is located in the −1 reading frame relative to ORF2.

Recent culture-independent sequencing studies of marine, freshwater, and other water-associated samples, from early metagenomic surveys [404,405,406,407,408,409] to more recent diverse analyses [410,411,412,413], have identified RNA virus-derived sequences homologous to members of the order *Picornavirales* (reviewed in [414]). These include dicistroviruses, iflaviruses, marnaviruses, picornaviruses, and secoviruses. Detailed analyses of metagenomically identified dicistrovirus-like viruses, including Halastavi árva RNA virus (HalV; NC_016418) [415], Nedicistrovirus (NediV; JQ898341) [407], and Wenling picorna-like virus 2 (Wenling 2; NC_032429) [409], have demonstrated that the IGR–IRESs encoded by these viruses mediate factorless translation initiation in reconstitution systems [393,416,417] —a hallmark of the family *Dicistroviridae*. Genetic divergence of ORF2 capsid gene sequences is evident among metagenomically identified dicistrovirus-like viruses (Figure 1 in Ref. [416]). This divergence may have coevolved with RNA structural elements in the IGR [393,416,417,418]. HalV IGR-IRES activity has recently been demonstrated in insect Sf21 cell lysates and *Drosophila* S2 cells [419].

Among these, four viruses in the HalV clade encode an extendable reading frame leading to ORF2. Variations in type II hexanucleotide sequences have been detected, including UGACUC in Shahe arthropod virus 1 (NC_032422) [409] and UGACGC in Changjiang picorna-like virus 14 (NC_032773) [409], Kuiper virus (KX657785), and Drellivirus (KX924637). The reading frame architecture of these viruses suggests that as described for PSIV [398], they have the potential to produce multiple polypeptides from a single RNA via readthrough and IGR–IRES-mediated translation, providing additional examples of noncanonical translation in eukaryotes.

#### 4.4.2. Triatoviral Extended Stem–Loop, Stem–Loop III (SLIII)

**Figure 7:**
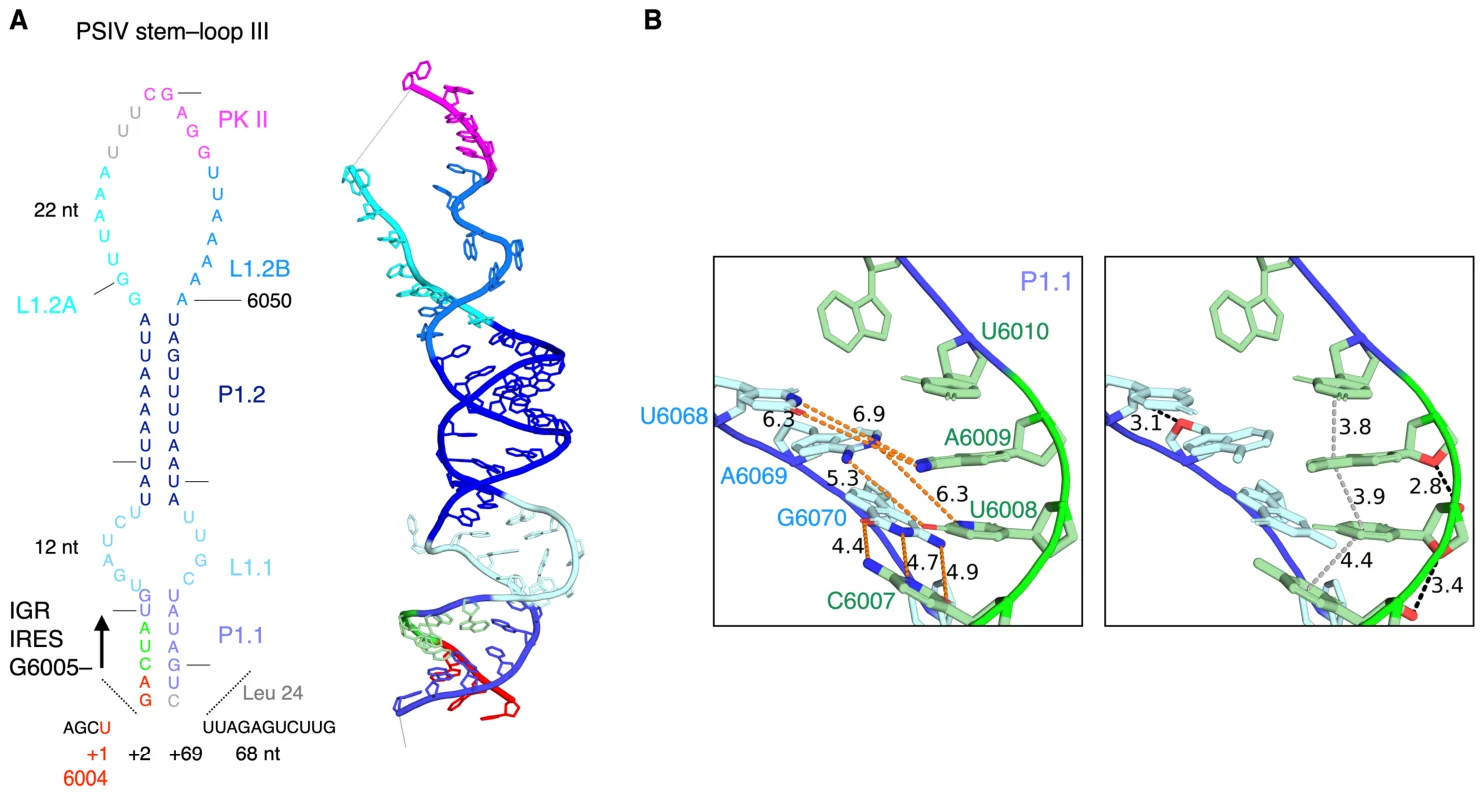
Downstream RNA structures for triatoviral UGA readthrough. (**A**) Primary sequence, predicted structural model, and X-ray crystal structures are shown for the triatovirus PSIV [421,424], with nucleotides U6036–U6038 and C6072–U6074 missing in the deposited coordinates (PDB ID: 2IL9), likely due to local disorder or flexibility. The stop-codon-related annotations follow those in Figure 5. Nucleotide positions follow the original viral numbering (e.g., 6010, 6020) used in previous studies. Amino acid numbering is based on the readthrough product, with the amino acid corresponding to the UGA stop codon designated as amino acid 1. (**B**) Distorted helical features of the P1.1 region observed in the RNA-only crystal of the PSIV IRES (2IL9). Distances between putative Watson–Crick base pairs (left, orange), base-stackings of the ascending strand (right, gray), and backbone hydrogen bonds (right, black) are shown. These measurements suggest that the P1.1 helix deviates from the canonical A-form geometry. Distances were measured in PyMOL, and structural geometry was analyzed using 3DNA/DSSR [425]. A6067 was omitted for clarity. IGR–IRES, intergenic region internal ribosomal entry site; PSIV, Plautia stali intestine virus.

Stimulatory effects of the downstream structure have not been observed in PSIV [69], as deletion of up to 183 nt from the 3′ end of the 186-nt IGR did not reduce readthrough activity. The optimality of the UGACUA hexanucleotide has been confirmed in a single-nucleotide replacement assay. Structural mapping using SHAPE reagents and base-modifying chemicals has ruled out the unlikely possibility that the most active sequence acquires stimulatory structure through interactions with the reporter or other portions of the transcript. Although structural mapping supports the formation of the P1.1 stem in SLIII, an RNA-only crystal structure with its downstream sequence reveals local distortion in this region [420,421] (Figure 7). The neighboring P1.2 stem remains close to an A-form helix, with only a subtle variation in base stacking, arguing against a global crystallization artifact. Backbone torsion angle deviations, likely influenced by the downstream 73-nt sequence, disrupt base pairing and increase inter-base distances, thereby destabilizing P1.1 and potentially weakening its function as a kinetic barrier.

Considering the need to sustain capsid protein production independently of upstream translation, together with the potential structural constraints between readthrough-inducing elements and IGR–IRES integrity, it is reasonable that the IGR–IRES structure does not strongly stimulate readthrough. The P1.1 segment from related dicistroviruses forms Watson–Crick base pairs in the 80S-bound [422,423] and subsequent factor-bound states [394], likely contributing to stabilization of the 60S–IRES interaction and providing a structural basis for efficient IGR–IRES-dependent capsid protein synthesis in dicistroviruses.

### 4.5. Mechanochemical Features of Downstream RNA Structures

Downstream RNA structures have been implicated in translational recoding events, including stop codon readthrough. Proposed roles include acting as barriers to ribosome progression, inducing ribosomal pausing, and facilitating interactions with *trans*-acting factors [62,320,426]. Although these mechanisms are not mutually exclusive, their relative contributions remain unclear. Comparative analysis across RNA viruses (Figure 4, Figure 5, Figure 6 and Figure 7) reveals both stimulatory RNA elements and cases in which readthrough occurs without structural requirements, underscoring the diversity of structural features involved.

The prominent feature is the spacing between the termination codon and the beginning of the structure, which is shared among MoMLV, VEEV, and CTFV and involves a stretch of consecutive G–C base pairs typically spanning three nucleotides. This characteristic may strengthen interactions between the structure and the ribosome entry pore as the termination codon enters the decoding center. An early pseudoknot reconstruction study in MoMLV, in which two out of three consecutive G–C pairs were replaced with A–U pairs, failed to restore readthrough efficiencies [76]. The accompanying reduction in stem stability may also have contributed to the observed loss of activity.

Measurements using optical tweezers (laser-based single-molecule techniques for measuring molecular forces and movements) have shown that elongating ribosomes require more time and force to traverse GC-rich or thermodynamically stable RNA hairpins [427,428], supporting this view. Consecutive G–C base pairs at the base of the structure are thermodynamically stable and likely function collectively as a barrier, thereby promoting ribosomal pausing, since elongating ribosomes require increased time and force to traverse such regions. Consistently, a silent mutation replacing a U–A pair in S1 with a C–G pair increases MoMLV readthrough efficiency twofold [99], whereas the reverse substitution reduces efficiency to a similar extent [97]. A similar effect has been reported for CTFV, where conversion of a central G:U wobble pair to a G–C pair increased readthrough efficiency [66]. Together, these observations support a role for proximal stem stability and GC content in modulating readthrough efficiency through the formation of a thermodynamic barrier.

mRNA spans approximately 15–17 nucleotides downstream from the P site in mammalian ribosomes [429,430] (Figure 6B in Ref. [429]). Consistently, approximately 11–12 nucleotides downstream of the A-site codon occupy the mRNA entry channel in elongating ribosomes [125,291] (Figure 3E). Spacers of eight nucleotides or longer in stimulatory structures (MoMLV, VEEV, SINV, and CTFV) likely position the downstream structure to interfere with or delay the normal progression of termination. Supporting this interpretation, shortening the spacer by one codon severely impaired readthrough efficiency [61,62]. The SARS-CoV-2 frameshifting structure, which begins at position +14 in the 80S ribosome–mRNA complex, together with the three-nucleotide translocation step of elongation (e.g., [399,428]), suggests that structures starting at position +12 may influence later stages of translocation, particularly middle to late stages. Progression delay may affect near-cognate elongation and normal termination but is likely to preferentially impair decoding during normal termination, as this process requires entry of the +4 nucleotide into the A site and ribosomal recognition of up to the +5 nucleotide through efficient stacking (Section 3.2.1 and Figure 3C,D). Near-cognate tRNAs provide additional opportunities to decode the termination codon during this delay, and its efficiency is increased by stimulatory elements, such as P-site–proximal environments, when encoded in the flanking sequence (kinetic trap model, Figure 1).

In contrast, the PSIV structure begins at the +2 nucleotide relative to the termination codon. Although the IGR–IRES extends up to 189 nt downstream of the termination codon, no stable secondary structure defined by consecutive G–C base pairs is observed near nucleotide position +12 (6015U in Figure 7A), despite the presence of a predominantly AU-rich P1.2 stem. This feature, together with the structural perturbation at the base of the P1.1 region (Figure 7B), is unlikely to promote readthrough.

Although CHIKV structures are related to those of VEEV, their stimulatory activities have not been clearly established. Notably, the predicted downstream structures differ from those of other stimulatory elements, lacking consecutive G–C base pairs and exhibiting shifted start positions (+9 or +13 instead of +12 or +16) (Figure 5C). Additionally, the hexanucleotide UGACUA at the readthrough site is known to support high basal readthrough. These structural factors and the high basal readthrough associated with the UGACUA motif may influence the observed readthrough activity.

Normal progression of translation elongation is proposed to involve at least two components: fluctuation-driven and force-driven [428]. The fluctuation-driven component reflects ribosomal conformational dynamics influenced by thermal fluctuations and shaped by local structural stability around the mRNA entry pore. In contrast, interactions with ribosomal outer surfaces, as observed in frameshifting structures [125,297], may counteract the force-driven component. Nucleotides in the single-stranded portion of the downstream structure can associate with ribosomal protein uS3 in *dnaX* (PDB 5UQ8), or with uS3 and eS10 in SARS-CoV-2 (Figure 3E), potentially constraining ribosomal movements required for normal translation. This restriction may be additionally modulated in SARS-CoV-2 by interactions involving ribosomal helix h16. Consistent with this idea in MoMLV, several nucleotides within the L2 region and the single-nucleotide L1 (A17) site contribute to readthrough efficiency, with certain mutations (e.g., U38A) exerting disproportionately strong effects (Section 4.1.3).

Modulation of thermodynamic stability, also referred to as structural integrity in some studies [97,99], can also counteract the force-driven component. Given that the functional principles of host ribosomes are evolutionarily conserved despite substantial structural divergence, viral downstream structures appear to employ multiple structural features to modulate normal ribosomal function, consistent with the kinetic trap model.

While the precise mechanisms through which these structures initiate inhibitory effects await experimental clarification, detailed investigations of individual viruses have provided insights into how downstream RNA elements modulate ribosome dynamics, with broader implications for cellular translation and host–virus interactions. Ultimately, these insights highlight RNA as an active regulator of ribosome dynamics, influencing translational outcomes beyond the canonical genetic code.

## 5. Future Directions and Concluding Remarks

Viral strategies and host countermeasures collectively shape stop codon readthrough efficiency by modulating decoding and related factors that engage the viral stop codon, as well as RNA structures that influence the ribosomal mRNA entry channel. The mechanistic basis of these interactions remains incompletely understood. The classical framework of kinetically separable phases proposed for decoding provides a useful conceptual basis for considering potential kinetic traps during translation (e.g., Figure 1A and Figure 3A,B). Recent structural studies suggest that these kinetically defined phases may encompass heterogeneous and dynamically interconverting conformational states rather than strictly discrete structural intermediates. Accordingly, integrating structural, kinetic, and mechanistic insights into the dynamic behavior of the ribosome will be important for refining mechanistic models of viral readthrough, including potential kinetic trap mechanisms.

### 5.1. Structural Perspective

At the structural level, interactions between downstream mRNA structures and entry pore proteins in eukaryotes (including uS3, uS5, and eS30; Figure 3E,F) can modulate the conformational sampling landscape and fidelity-related transitions in the decoding center that are required for progression to GTPase activation. uS3, located on the opposite side of h16, contributes to shaping the mRNA entry channel and forms mRNA contacts that are consistently observed across structurally and functionally distinct ribosomal states, including 48S initiation complexes [431,432], translation-stalling collision complexes [433,434,435], and IGR–IRES-mediated initiation complexes [312,393]. These observations collectively support the conserved role of uS3 in the ribosomal head region as a gatekeeper for incoming mRNA. In contrast, evidence for mRNA interactions with the eukaryote-specific protein eS30 in the ribosomal shoulder region remains limited, with clear examples observed in SARS-CoV-2 pseudoknot-mediated recoding [125]. While the N-terminal extension of eS30, which reaches toward the decoding center (Figure 3F), may influence canonical elongation, more proximal segments interact with uS12, 18S rRNA h18, and uS4 near the mRNA entry pore (Figure 3E). When stabilization of canonical base pairs is affected, eS30–mRNA downstream interactions may propagate structural changes from the ribosomal shoulder toward the decoding center, potentially influencing domain closure and termination codon recognition. How these structural constraints are balanced to yield readthrough remains incompletely understood.

Notably, recent structural findings have identified the RNA helicase DHX29 as being associated with stalled ribosomes bearing a UAA termination codon, where it contacts proximal eS30 residues (Pro91 and Lys92) and uS12, within the region linking the decoding center and the mRNA entry channel in the human 80S ribosome (PDB 22TU [126]; representative contacts are shown in Figure 3E). However, its functional contribution to translational readthrough remains unclear. Collectively, these observations highlight the potential importance of this region in controlling decoding kinetics through structural perturbation. Whether additional *trans*-acting factors contribute to the regulation of decoding kinetics within this region remains an open question.

### 5.2. Mechanistic Insights

At the mechanistic level, structural, kinetic, and single-molecule approaches, together with molecular dynamics simulations, have provided insights into the rejection of near-cognate tRNAs during elongation (Figure 3A). Based on these advances, a recent modeling study in yeast revealed a role for elongation-time shaping in tRNA selection [436]. Regarding stop codon decoding, experimentally determined efficiencies in both eukaryotes (e.g., Table 3) and bacteria can exceed 10% in some studies. These observations suggest that a measurable fraction of near-cognate tRNAs proceeds through both initial selection and kinetic proofreading steps under specific conditions. Increased readthrough efficiency at thermodynamically favorable UGA or UAG stop codons (Section 3.3.1, (2) Stop Codon Identity) compared with UAA (Section 3.3.1, (2) (ii) UAA (ochre) Mutations, NMD, and Viral Replication) suggests that kinetic proofreading can contribute to stop codon readthrough. Consistent with this interpretation, recent single-molecule studies have shown that near-cognate tRNA trajectories can transiently populate accommodated states during repeated sampling events prior to peptide release, providing a dynamic view of kinetic proofreading at the single-molecule level [128]. Observations involving pseudouridine-modified base pairs may additionally support this interpretation, although their effects could arise during either initial selection or kinetic proofreading. Additional support may come from fidelity-related effects of tRNA modifications such as MIA37, which have been reported to influence near-cognate decoding in *E. coli* (Section 3.3.1, (2) (i) Near-cognate tRNAs and Kinetic Proofreading). Originally proposed as a kinetic mechanism to increase decoding fidelity, proofreading compensates for the rejection of near-cognate tRNAs that escape initial selection, as supported by codon-dependent experimental evidence from bacteria [117,437], with fidelity theoretically linked to codon-specific rate constants [438]. Eukaryotic ribosomal errors are estimated to be lower than those in bacteria, suggesting that proofreading in eukaryotes may be more active and elaborate. The decoding center governs specificity in distinguishing cognate from near-cognate base pairs, whereas global tRNA–ribosome interactions modulate stability and dwell time. Together, these factors are thought to shape the transition of tRNA from the pre-accommodation A/T state to the accommodated A/A state, although the contribution of global ribosomal dynamics to this process remains poorly characterized. Here, accommodation is used in a broad sense, extending from the factor rearrangement phase (up to eEF1A–GDP release) through to the tRNA transition from the A/T state to the fully accommodated A/A state (Figure 3A).

As A–minor interactions are observed during proofreading in bacterial ribosomes (*E. coli* 70S [138]) and similar interactions are stabilized by eS30 hydrogen bonding in eukaryotic accommodated states (human 80S 8G61 [119], rabbit 80S 9YPY [133]; see also Figure 3F), factors governing initial selection at the decoding center continue to influence near-cognate selection. Additional factors, including tRNA conformational transitions during accommodation and structural coupling to the peptidyl transferase center, may further influence proofreading fidelity [145,149,438]. In this context, determinants of ribosomal kinetic proofreading, driven by GTP hydrolysis on eEF1A, likely extend beyond the decoding center to encompass how the energy is transmitted through the tRNA and ribosome, including subunit dynamics. Time-resolved cryo-EM provides structural snapshots corresponding to distinct accommodation states, whereas single-molecule approaches and simulations are required to resolve the kinetic and dynamic pathways connecting these states. Ribosomal rolling dynamics [146,147], although not unique to eukaryotes, may involve more pronounced tilting (i.e., deviation of the rotational axis, observed across both eukaryotic and bacterial ribosomes) [439] and are likely coupled to kinetic proofreading [149], further underscoring the need for an integrative framework across methods.

In animal virus readthrough, near-cognate elongation reflects a dynamic balance between selection and rejection pathways, which is ultimately shaped by the coupled structural and kinetic properties of the ribosome. As described above (Section 5.1), downstream stimulatory structures can modulate the decoding landscape, potentially through interactions with eS30 and related factors, and these effects may extend into the proofreading phase. In contrast, readthrough events that rely less extensively on downstream stimulatory RNA structures, such as those associated with the UGACUA hexanucleotide (type II), may provide a simplified model for dissecting the mechanistic contributions of decoding and proofreading steps.

### 5.3. Concluding Remarks

In conclusion, studies of RNA elements mediating translational readthrough in diverse viruses have provided valuable insights into the molecular mechanisms underlying stop codon readthrough. Biochemical and molecular biology approaches, including mutagenesis, reporter assays, and structural probing of RNAs, offer versatile tools for dissecting these mechanisms in individual viral systems. Importantly, it has become clear that host cells also exploit stop codon readthrough in processes such as metabolic regulation and stress responses (Section 3.1.1), as exemplified by the yeast prion [*Psi^+^*] phenotype [440], in which reduced translation termination fidelity increases stop codon readthrough [441], and by the broader regulatory roles of stop codon readthrough in gene expression [4]. Termination is not merely a strict endpoint of translation but can serve as a regulatory node at which multiple alternative outcomes arise. While viruses exploit readthrough and related mechanisms to expand coding capacity, similar principles operate in cellular systems, where translation termination efficiency can be modulated under specific conditions, potentially favoring continued translation over new translation initiation events, particularly in eukaryotic systems where initiation is energetically demanding. These parallels suggest that viral strategies may reflect the exploitation of intrinsic translational plasticity in host cells, rather than entirely distinct mechanisms.

## Figures and Tables

**Table 2:** Animal viruses with reported stop codon readthrough sequences.

Family	Genus	Complex/ Host Range	Species Name ^a,b,c,d^	Number of Species (Full-Length Genome Sequenced) ^e^
*Retroviridae*	*Gammaretrovirus*	–	**BaEV**, CrERV, **FeLV**, **GaLV**, HEMV, KoRV, McERV, MDEV, **MoMLV**, **MoMSV** ^a^, MuLV ERV ^a^, OOEV, PERVA, PERVB, PERVC, RD114, **REV** ^a^, RfRV, WMSV	19
	*Epsilonretrovirus*	–	WDSV	1
	Unclassified retroviruses	–	SnRV, SSSV, two endogenous retroviruses (RMEV; ZFERV)	4
*Togaviridae*	*Alphavirus*	VEE	**VEEV**	1
		EEE	EEEV	1
		WEE	AURAV ^b^, FMV, HJV, **SINV** ^b^, WEEV, WHATV	6
		SF	BEBV, **CHIKV** ^b^, GETV ^b^, MAYV, ONNV ^b^, RRV ^b^, SFV ^b^, UNAV	8
		Others	AHPV, BFV ^b^, CAAV, MIDV, NDUV, SESV, TROV	7
		insect-specific	ASALV, EILV, MWAV, TALV, YYV	5
		fish	Wenling fish alphavirus	1
*Reoviridae*	*Coltivirus*	–	CHCV ^c^, **CTFV**, EYAV, KUNDV, TarTV, TFRV	6
*Dicistroviridae*	*Triatovirus*	–	**PSIV** (TrV) ^d^	1

Virus names and abbreviations generally follow ICTV nomenclature where available; otherwise, commonly used names are retained. In this analysis, alphaviruses are grouped according to major antigenic relationships and host range. Abbreviations: AHPV, Alaskan harbor porpoise virus; ASALV Agua Salud alphavirus; AURAV, Aura virus; BaEV, baboon endogenous virus; BEBV, bebaru virus; BFV, Barmah Forest virus; CAAV, Caainguá virus; CHCV, California hare coltivirus; CHIKV, chikungunya virus; CrERV, cervid endogenous gammaretrovirus; CTFV, Colorado tick fever virus; EEEV, eastern equine encephalitis virus; EILV, Eilat virus; EYAV, Eyach virus; FeLV, feline leukemia virus; FMV, Fort Morgan virus; GaLV, gibbon ape leukemia virus; GETV, getah virus; HEMV, *hortulanus* endogenous murine leukemia virus; HJV, Highlands J virus; KoRV, koala retrovirus; KUNDV, Kundal virus; MAYV, Mayaro virus; McERV, *Mus caroli* endogenous virus; MDEV, *Mus dunni* endogenous virus; MIDV, Middelburg virus; MoMLV, Moloney murine leukemia virus; MoMSV, Moloney murine sarcoma virus; MuLV ERV, mouse leukemia virus endogenous retrovirus; MWAV, Mwinilunga alphavirus; NDUV, Ndumu virus; ONNV, o’nyong-nyong virus; OOEV, Orcinus orca endogenous retrovirus; PERV, porcine endogenous retrovirus; PSIV, Plautia stali intestine virus; RD114, feline RD114 retrovirus; REV, reticuloendotheliosis virus; RfRV, *Rhinolophus ferrumequinum* retrovirus; RMEV, Rhinella marina endogenous retrovirus; RRV, Ross River virus; SESV, southern elephant seal virus; SFV, Semliki Forest virus; SINV, Sindbis virus; SnRV, snakehead retrovirus; SSSV, Atlantic salmon swim bladder sarcoma virus; TALV, Taï Forest alphavirus; TarTV, Tarumizu tick virus; TFRV, Täi forest reovirus; TROV, Trocara virus; TrV, Triatoma virus; UNAV, Una virus; VEEV, Venezuelan equine encephalitis virus; WDSV, walleye dermal sarcoma virus; WEEV, western equine encephalitis virus; WHATV, Whataroa virus; WMSV, woolly monkey sarcoma virus; YYV, Yada Yada Virus; ZFERV, Danio rerio endogenous retrovirus. ^a^ Species with full-length sequences are listed. Viruses in bold have experimentally verified stop codon readthrough (see Table 1 and Table 3). MoMSV: Full-length (5833 nt) replication-defective gammaretrovirus requiring a helper murine leukemia virus for replication. MuLV ERVs: Full-length endogenous gammaretroviruses in laboratory mice. Although listed as a single species here, MuLV ERVs include viruses with different *env* genes distributed across more than 50 genomic loci [80]. REV: Spleen necrosis virus is classified within the reticuloendotheliosis virus (REV) group. ^b^ Some *Alphavirus* species contain a sense codon at their readthrough UGA site. These are AURAV (CGA) and SINV (UGU) in the western equine encephalitis (WEE) complex; CHIKV (AGA or CGA), GETV (AGA), ONNV (CGA, MGA, or UGG), RRV (CGA, UGC, or UGU), and SFV (CGA) in the Semliki Forest (SF) complex; and BFV (UGG) in the Barmah Forest (BF) complex. ^c^ CHCV is considered a distinct species in this review (Section 4.3.1), resulting in a total of six recognized species in the genus *Coltivirus*. ^d^ TrV contains a reading frame spanning the entire IGR downstream of ORF1; readthrough has not been experimentally demonstrated. ^e^ Number of viral species containing a readthrough site, counted at the genus level, at the alphaviral complex level for alphaviruses, or as unclassified retroviruses where applicable.

## Data Availability

No new data were created or analyzed in this study. Data sharing is not applicable to this article.

## Abbreviations

AHPV, Alaskan harbor porpoise virus; AURAV, Aura virus; BaEV, baboon endogenous virus; CAAV, Caainguá virus; CHCV, California hare coltivirus; CHIKV, chikungunya virus; CIRV, carnation Italian ringspot virus; CTFV, Colorado tick fever virus; CrPV, cricket paralysis virus; EEEV, eastern equine encephalitis virus; EILV, Eilat virus; EYAV, Eyach virus; FeLV, feline leukemia virus; FMV, Fort Morgan virus; GaLV, gibbon ape leukemia virus; HalV, Halastavi árva virus; HCMV, human cytomegalovirus; HIV-1, human immunodeficiency virus 1; IAPV, Israeli acute bee paralysis virus; IBV, infectious bronchitis virus; KoRV, koala retrovirus; MDPV, Mosso das Pedras virus (VEEV-IF); MMTV, mouse mammary tumor virus; MoMLV, Moloney murine leukemia virus; ONNV, o’nyong-nyong virus; OPRL1, opioid related nociceptin receptor 1; PSIV, Plautia stali intestine virus; REV, reticuloendotheliosis virus; RNV, Rio Negro virus (VEEV VI); RRV, Ross River virus; SARS-CoV-2, severe acute respiratory syndrome coronavirus 2; SAV, salmonid alphaviruses; SDV, sleeping disease virus; SFV, Semliki Forest virus; SINV, Sindbis virus; SnRV, snakehead retrovirus; SPDV, salmon pancreatic disease virus; SSSV, Atlantic salmon swim bladder sarcoma virus; TFAV, Taï Forest alphavirus; TMV, tobacco mosaic virus; TROV, Trocara virus; TrV, Triatoma virus; TSV, Taura syndrome virus; VEEV, Venezuelan equine encephalitis virus; WDSV, walleye dermal sarcoma virus; WEEV, western equine encephalitis virus; Wenling 2, Wenling picorna-like virus 2; WHATV, Whataroa virus; ZFERV, Danio rerio endogenous retrovirus.
